# Quantitative trait locus mapping of osmotic stress response in the fungal wheat pathogen *Zymoseptoria tritici*

**DOI:** 10.1093/g3journal/jkad226

**Published:** 2023-09-29

**Authors:** Jessica Stapley, Bruce A McDonald

**Affiliations:** Plant Pathology Group, Institute of Integrative Biology, ETH Zurich, Zürich 8092, Switzerland; Plant Pathology Group, Institute of Integrative Biology, ETH Zurich, Zürich 8092, Switzerland

**Keywords:** salt stress, plant pathogen, fungi, *Mycosphaerella graminicola*, QTL

## Abstract

Osmotic stress is a ubiquitous and potent stress for all living organisms, but few studies have investigated the genetic basis of salt tolerance in filamentous fungi. The main aim of this study was to identify regions of the genome associated with tolerance to potassium chloride (KCl) in the wheat pathogen *Zymoseptoria tritici.* A secondary aim was to identify candidate genes affecting salt tolerance within the most promising chromosomal regions. We achieved these aims with a quantitative trait locus (QTL) mapping study using offspring from 2 crosses grown in vitro in the presence or absence of osmotic stress imposed by 0.75 M KCl. We identified significant QTL for most of the traits in both crosses. Several QTLs overlapped with QTL identified in earlier studies for other traits, and some QTL explained trait variation in both the control and salt stress environments. A significant QTL on chromosome 3 explained variation in colony radius at 8-day postinoculation (dpi) in the KCl environment as well as colony radius KCl tolerance at 8 dpi. The QTL peak had a high logarithm of the odds ratio (LOD) and encompassed an interval containing only 36 genes. Six of these genes present promising candidates for functional analyses. A gene ontology (GO) enrichment analysis of QTL unique to the KCl environment found evidence for the enrichment of functions involved in osmotic stress responses.

## Introduction

Osmotic stress is a common and potent environmental stress that can limit growth and reproduction in many organisms. At the cellular level, osmotic stress results in changes to the movement of water into or out of the cell through osmosis. To counteract osmotic stress, organisms need to accumulate compatible solutes (e.g. glycerol, erythritol, arabitol, mannitol, and trehalose) that can balance osmosis and provide turgor pressure (Kogej *et al.* 2007; Araújo *et al.* 2020). In fungal pathogens of plants, the pathogen may be exposed to a relatively high salt concentration (0.5 M) within the plant (see Araújo *et al.* 2020) as well as experience changes in salt and osmolyte concentrations as plant tissues degrade (Jacob *et al.* 2015). The genes and pathways involved in responding to the resulting osmotic stresses may also affect plant–pathogen interactions and virulence. For example, turgor pressure is mediated by changes in vacuole size, and the number is important in both plant tissue penetration and response to osmotic stress (Richards *et al.* 2010). Genes involved in cell wall integrity and remodeling, e.g. glycoside hydrolases, can mediate osmotic stress response and virulence (Li *et al.* 2016). Voltage-gated chloride channels, which are involved in the regulation of cell volume, also influence virulence in fungal pathogens of both plants and humans (Zhu and Williamson 2003; Cañero and Roncero 2008). Knowledge of the genetic basis of osmotic sensitivity in plant pathogens can thus provide insights into the genetic basis of environmental stress responses as well as disease development.

The immediate effects of osmotic stress in fungi include stalled growth, changes in the recruitment of water, collapse of the cytoskeleton, altered plasma membranes, and a reduction in glycerol transport (Hohmann 2002; Duran *et al.* 2010). Longer-term adaptation to osmotic stress involves modifications of metabolism, plasma membranes, cell wall thickness, and cell wall melanization, as well as an accumulation of mycosporines (Hohmann 2002; Kogej *et al.* 2007). Osmotic stress is also likely to activate many genes and pathways involved in the “general stress response.” For example, up to 10% of the genes that were differentially expressed under different individual stressors were shared across different osmotic stress treatments (Hohmann 2002). Stress response regulators and genes such as HSP90 and GTPase *Ras1* are considered promising antifungal drug targets for human pathogens (LeBlanc *et al.* 2020). These proteins may also become fungicide targets for plant pathogens as our understanding of the molecular mechanisms governing stress responses in plant pathogenic fungi improves.

Although the key salt stress response pathways and genes have been identified and well characterized in model yeasts, the pathways in filamentous fungi are still poorly understood. However, they could provide novel insights into the genes involved in responding to osmotic stress, into plant–pathogen interactions, and fungicide resistance (Jacob *et al.* 2015). The high-osmolarity glycerol (HOG) pathway is an osmoresponsive system and one of the most well-understood mitogen-activated protein (MAP) kinase pathways (Hohmann 2002). In yeast, the upstream pathway of HOG has 2 independent signaling branches (Sln1-branch and Sho1-branch) that sense osmotic stress differently. These pathways converge on the MAP kinase, *Pbs2*, which phosphorylates *Hog1p* (de Nadal and Posas 2022). In plant pathogens, upstream HOG signaling appears more complex than in yeast, although it is still poorly understood (Jacob *et al.* 2015). In *Saccharomyces cerevisiae* on the Sln1-branch, there is only 1 histidine kinase (HK) (Sln1p) (Jacob *et al.* 2015), whereas several filamentous pathogenic fungi, including our model *Zymoseptoria tritici*, have multiple (up to 17) HKs (Jacob *et al.* 2015). Several studies have identified transcription factors (TFs) involved in regulating the fungal response to salt stress, as well as other stressors—including temperature and fungicide stress. For example, in *Aspergillus oryzae*, GATA TFs were shown to be important in responding to salt and temperature stress (Jiang *et al.* 2021). In the causal agent of Fusarium head blight (*Fusarium graminearum*), the nutrient and stress factor 1 (NSf1) C_2_H_2_ zinc fingers protein plays an important role in salt stress response, sexual–asexual reproduction, vegetative growth, fungicide sensitivity, and pathogenicity (Shi *et al.* 2021), and a Zn(2)-C6 fungal-type DNA-binding TF (Fss1) is required for sodium and lithium tolerance (Son *et al.* 2015). Cell wall melanization also appears to confer benefits for osmotic stress tolerance. For example, cell wall melanization in halotolerant fungi helps to maintain high concentrations of glycerol and contain the resulting turgor pressure by strengthening and preventing leakage through the cell wall (Kogej *et al.* 2007). These examples coming from a relatively small number of fungal species demonstrate how the molecular mechanisms of salt response can vary across taxa and illustrate that additional work is needed to understand how filamentous fungi adapt to salt stress.

To improve our understanding of osmotic stress response in fungi, we performed a quantitative trait locus (QTL) mapping study using the wheat pathogen *Z. tritici* growing under salt stress. QTL studies have been instrumental in finding candidate genes for multiple traits in this widespread and damaging wheat pathogen, including melanization (Lendenmann *et al.* 2014), fungicide sensitivity (Lendenmann *et al.* 2015), morphological switching (Francisco *et al.* 2023), and oxidative stress (Zhong *et al.* 2021), and enabled the cloning and functional validation of genes affecting virulence (Zhong *et al.* 2017), melanization (Krishnan *et al.* 2018), and morphological switching (Francisco *et al.* 2023). QTL mapping identifies genomic regions that explain variation in a trait and that are likely to harbor genes responsible for creating variation in the trait. The aim of this study was to identify large-effect loci associated with tolerance to salt stress in *Z. tritici* and identify plausible candidate genes within the most promising QTL intervals.

## Methods

### QTL mapping crosses

Parent and progeny isolates used in this study are from 2 crosses that were described previously (Lendenmann *et al.* 2014). The original choice of strains for making crosses was based upon a comprehensive phenotyping campaign of an international collection of 150 strains, including 30 strains from Switzerland (Zhan *et al.* 2005). In this original phenotyping campaign, we measured 7 traits for each strain, including in planta virulence and pycnidium size and density, and growth rates in vitro under different temperatures and in the presence of fungicides. Based on data from these 7 traits, 4 Swiss strains were selected to make the QTL mapping crosses that yielded 700 offspring over the 2 crosses. The 4 parents have since been tested under other environmental stressors (e.g. reactive oxygen; Zhong *et al.* 2021) including potassium chloride (KCl) (0.25–1.0 M). In the presence of 0.75 M KCl, the parents differed in both growth and melanization traits (unpublished data). The crosses were made by coinfecting wheat leaves with the parent strains, with the resulting ascospores collected from infected leaves and grown in vitro. One cross was between ST99CH1A5 and ST99CH1E4 (herein referred to as 1A5 and 1E4, respectively), and the other was between ST99CH3D7 and ST99CH3D1 (herein referred to as 3D7 and 3D1, respectively).

### Genotyping

SNP genotype data for the progeny were obtained from a RAD sequence (RADseq) data set that was previously produced in our lab (first described in Lendenmann *et al.* 2014). In brief, the genome was cut using the restriction enzyme *Pst*l, and the libraries were sequenced on an Illumina HiSeq2000 with paired-end sequencing. Complete genome sequences of the parental strains (Croll *et al.* 2013) [NCBI Biosample: SRS383146 (ST99CH3D1), SRS383147 (ST99CH3D7), SRS383142 (ST99CH1A5), and SRS383143 (ST99CH1E4)] were used to SNP genotype the parents and offspring.

RADseq processing and variant discovery are described in detail at https://github.com/jessstapley/QTL-mapping-Z.-tritici. In brief, RADseq reads were trimmed of adaptors and low-quality sequence using trimmomatic (v0.35). The RADseq reads were mapped to a reference genome using bwa mem (v0.7.17). Reads from the progeny of the 3D7 × 3D1 cross were mapped to the 3D7 reference genome, and reads from the progeny of the 1A5 × 1E4 cross were mapped to the 1A5 reference genome. Variant calling was done using the GATK Germline Short Variant Discovery pipeline, following their Best Practice recommendations (https://gatk.broadinstitute.org/hc/en-us/sections/360007226651-Best-Practices-Workflows). After this, we applied the following filters: only biallelic SNPs, parents had alternative alleles, <50% missing genotypes per marker, <50% missing genotypes per individual, mean read depth of >3 and <30, depth quality (QD) of >5, mapping quality of >40, and minor allele frequency of >0.02 and <0.80, and SNPs in regions where the SNP density is >3 in 10 bp were removed.

### Generating linkage maps

Linkage maps were made using R (R Core Team 2020), R Studio (RStudio Team 2020), and the R package qtl (v1.48-1, Broman *et al.* 2003). As the order and base pair positions of the markers were known from the reference genomes, we only needed to calculate the recombination distance between markers to create the linkage map. Before map construction, we removed putative clones. To do this, we calculated genetic similarity using the “comparegeno” function and removed individuals with >99% shared genotypes (83 progenies were removed from the 1A5 × 1E4 cross, and 74 progenies were removed from the 3D7 × 3D1 cross). The linkage map was constructed for each chromosome separately using the “estimatemap” function with the method “morgan.” Then, we performed multiple rounds of cleaning to remove error-prone markers that caused map inflation. First, we removed markers that had an estimated recombination distance above the 99.9% quantile. Then, the recombination rate was estimated again, and we inspected the maps. In regions of the map where there was evidence of map inflation and spurious double recombination events, we visualized these regions in an Integrated Genome Viewer (IGV, https://igv.org) to look at the mapping alignments. Markers were removed from regions with poor alignments and evidence of mismapping. A summary of the 2 linkage maps is provided in Supplementary Table 1, and the complete maps are available online (https://github.com/jessstapley/QTL-mapping-Z.-tritici). After applying all of these filters, the average marker spacing was 1,217 (0.19 cM) and 529 bp (0.14 cM) for the 1A5 × 1E4 and 3D7 × 3D1 crosses, respectively.

### Phenotyping

We compared colony growth and melanization in a control environment and in the presence of KCl to simulate a salt stress environment. KCl has been used extensively in the study of stress responses in fungi because it is environmentally ubiquitous and lacks the toxic effects of Na^+^ (Hohmann 2002; Araújo *et al.* 2020). Only nonclonal offspring strains were phenotyped (for 1A5 × 1E4 *n* = 259 strains; for 3D7 × 3D1 *n* = 265 strains). The basic protocols for isolate recovery from −80°C storage, growth in vitro, and the measurements of colony size and colony melanization were described previously (Zhong *et al.* 2021). In brief, Petri dishes containing Difco potato dextrose agar (PDA) were inoculated with 200 µl of a spore solution (concentration 200 spores/ml) and grown at 18°C for 8 and 12 days. In the salt stress environment, a sterile KCl solution was added to the cooled and sterilized PDA to make a final concentration of 0.75 M. Three replicates were grown in both the control and salt (KCl) environments.

Two traits were measured; colony area and colony gray value, using automated image analysis as described previously (Lendenmann *et al.* 2014; Zhong *et al.* 2021). Gray value was measured on a scale of 0–255, where darker, more melanized colonies have lower values (0 = black, 255 = white). Mean colony area (mm^2^) and gray value were calculated from measurements of multiple colonies on each plate, and then the mean was calculated across the 3 replicate plates to obtain a final mean colony area and mean gray value for each strain in the 2 environments. The colony area was converted to colony radius by dividing by π and taking the square root of this value. Measurements were taken at 8- and 12-day postinoculation (dpi). For the analysis, we used 3 different types of data: (1) time point/age: the colony radius and gray value at 8 and 12 dpi; (2) daily rate measurements: the change in colony radius or gray value between 8 and 12 dpi (i.e. the difference in colony radius between 8 and 12 dpi/difference in days); and (3) KCl tolerance measurements: the ratio of a measurement (colony radius, gray value, growth rate, or melanization rate) between the KCl environment and the control environment (i.e. colony radius KCl tolerance at 8 dpi = colony radius at 8 dpi in the KCl environment/colony radius at 8 dpi in the control environment, or growth rate KCl tolerance = growth rate in the KCl environment/growth rate in the control environment). For the KCl tolerance measurements, values > 1 indicate that for that trait, an isolate is more tolerant to KCl (higher value under salt stress), and values < 1 indicate that for that trait measured, an isolate is more sensitive to KCl. In total, there were 18 traits (Table 1).

**Table 1.** Summary of the QTL mapping results for all traits.

Data type	Environment	DPI	Trait	Trait full name	QTL peak 3D7 × 3D1	*h* ^2^	QTL peak 1A5 × 1E4	*h* ^2^
Time point	Control	8	Colony radius	Colony radius in control at 8 dpi	3, 8, 10, 11	0.53	2, 3, 8	0.56
Time point	Control	12	Colony radius	Colony radius in control at 12 dpi	3, 7, 8, 10, 11	0.62	7, 8	0.48
Time point	KCl	8	Colony radius	Colony radius in KCl at 8 dpi	3, 4	0.64	6	0.50
Time point	KCl	12	Colony radius	Colony radius in KCl at 12 dpi	3, 8	0.60	1, 8	0.52
Time point	Control	8	Gray value	Gray value in control at 8 dpi	8, 10, 11	0.42	1, 2, 3, 8	0.63
Time point	Control	12	Gray value	Gray value in control at 12dpi	11	0.22	2, 3, 8	0.44
Time point	KCl	8	Gray value	Gray value in KCl at 8 dpi	8, 11	0.54	8	0.34
Time point	KCl	12	Gray value	Gray value in KCl at 12dpi	1, 2, 11	0.34	No Sig. QTL	0.29
Rate	Control	8–12	Growth rate	Growth rate in control	3, 8, 11	0.61	7,8	0.33
Rate	KCl	8–12	Growth rate	Growth rate in KCl	3, 8, 11	0.45	8,	0.49
Rate	Control	8–12	Melanization rate	Melanization rate in control	10	0.28	2, 3, 12	0.54
Rate	KCl	8–12	Melanization rate	Melanization rate in KCl	1, 6	0.32	No Sig. QTL	0.27
Tolerance	Both	8	Gray value	Gray value KCl tolerance at 8 dpi	8, 10	0.37	1, 2, 3, 8	0.58
Tolerance	Both	12	Gray value	Gray value KCl tolerance at 12 dpi	11	0.18	No Sig. QTL	0.34
Tolerance	Both	8	Colony radius	Colony radius KCl tolerance at 8 dpi	3, 4, 10	0.59	8	0.42
Tolerance	Both	12	Colony radius	Colony radius KCl tolerance at 12 dpi	3, 10, 11	0.47	1	0.37
Tolerance	Both	8–12	Growth rate	Growth rate KCl tolerance	11	0.40	1, 5	0.45
Tolerance	Both	8–12	Melanization rate	Melanization rate KCl tolerance	1	0.14	No Sig. QTL	0.32

Gray value and colony radius were measured at 2 time points: 8 and 12 dpi. Growth rate was calculated as the change in colony radius/number of days. Melanization rate was calculated as the change in gray value/number of days. Colony radius and gray value KCl tolerance were calculated by dividing the value (gray value or colony radius) in the control environment by the value in the KCl environment. Growth rate and melanization KCl tolerance were calculated by dividing the rate (melanization or growth) in the control environment by the rate in the KCl environment. The QTL peak columns show the chromosomal position of significant QTL peak for each trait in the 3D7 × 3D1 and 1A5 × 1E4 crosses, respectively, and the final column contains estimated heritability (*h*^2^) for each trait.

### QTL mapping and analysis of genes within QTL intervals

QTL mapping was performed using the R (v 3.6.0) package qtl2” (v2_0.24) as described in detail on GitHub (https://github.com/jessstapley/QTL-mapping-Z.-tritici). We scanned the genome for a single QTL per chromosome with the “*scan1*” function using a linear mixed effect model, and we controlled for the relatedness of individuals (i.e. we included a random polygenic effect) by incorporating the kinship matrix into the model. Models that take into account the genetic covariance between individuals can reduce the false discovery rate in QTL scans and outperform models that do not include this information in the model (Malosetti *et al.* 2011). The significance threshold for a QTL peak was determined by permutation tests (*n* = 1,000), and we calculated a Bayes credible interval (95%) to identify the interval size around the QTL peak. We used custom R scripts to extract a list of the genes within the interval from the annotation (gff) files of each reference genome. The putative encoded function of each gene was determined in previous analyses (for 1A5, see Plissonneau *et al.* 2018; for 3D7, see Plissonneau *et al.* 2016). The annotations were obtained using InterProScan (https://www.ebi.ac.uk/interpro/) against multiple protein databases and then screened using multiple methods (e.g. Signal, https://services.healthtech.dtu.dk/service.php? SignalP-5.0; Phobius, https://phobius.sbc.su.se/) to determine if the encoded proteins contained likely signal peptides or secreted domains. The putative effect of a SNP in the coding sequence was determined using SNPeff (http://pcingola.github.io/SnpEff/). Pathway [gene ontology (GO) and Kyoto Encyclopedia of Genes and Genomes (KEGG)] enrichment analysis was performed for each trait with a significant QTL that was unique to the KCl environment following a tutorial for nonmodel species (https://archetypalecology.wordpress.com/2021/01/27/how-to-perform-kegg-and-go-enrichment-analysis-of-non-model-species-using-r/). GO annotations were retrieved from the annotation files describe above. For GO enrichment, the R package “topGo” (Alexa and Rahnenfuhrer 2023) was used to perform a Fischer test on the genes within each QTL interval following the guidelines in the vignette. All 3 ontologies—biological process (BP), cellular component (CC), and molecular function (MF), were analyzed. For KEGG pathways enrichment, GhostKOALA (https://www.kegg.jp/ghostkoala/) was used to find K numbers for all transcripts. The program found K numbers for 34% (4,091/11,726) and 49% (5,970/12,072) of transcripts for 3D7 and 1A5, respectively. The enrichment test was performed using the R package clusterProfiler (Wu *et al.* 2021) and the “enricher” function. The background set for both GO and KEGG was the entire gene/transcript set for each reference genome (3D7 and 1A5).

### Identifying putative candidate genes in the 3D7 × 3D1 cross

Genes within a single QTL interval on chromosome 3 were further investigated to identify the most likely candidate genes responsible for the phenotype. We identified the orthologous genes in the 3D1 parent using an analysis performed across the genomes of 19 reference strains (Badet *et al.* 2020). We inspected the alignments in the region of interest in IGV. We also blasted the 3D1 gene sequences (including ±200-bp flanking regions) against the 3D7 genome to calculate sequence similarity and identify indels.

### RNAseq data analysis

We analyzed RNAseq data that were previously created for the 4 parental strains during infection of the susceptible wheat cultivar Drifter (Palma-Guerrero *et al.* 2017) as well as cultures grown in vitro on 2 types of liquid media [yeast sucrose broth (YSB): yeast extract 10 g/L and sucrose 10 g/L, pH 6.8; and carbon-depleted minimal medium (MM), pH 5.8; Francisco *et al.* 2019]. The raw sequence reads were downloaded from the Short Read Archive (SRA, https://www.ncbi.nlm.nih.gov/sra, Bioproject: *in vitro* SRP152081 and *in vivo* SRP077418). RNAseq data processing and analysis are described in detail at GitHub (https://github.com/jessstapley/QTL-mapping-Z.-tritici). In brief, reads were trimmed and then mapped to 1 of 2 reference genomes (reads from 3D1 and 3D7 were mapped to the 3D7 reference genome while reads from 1E4 and 1A5 were mapped to the 1A5 reference genome). Then, the number of reads mapping to each gene was counted using R::Rsubread (Liao *et al.* 2019), and we tested for differential gene expression between strains, 3D7 vs 3D1 and 1A5 vs 1E4, using R::EdgeR (Robinson *et al.* 2010).

## Results

### Effect of KCl on growth and melanization

**Fig. 1.**
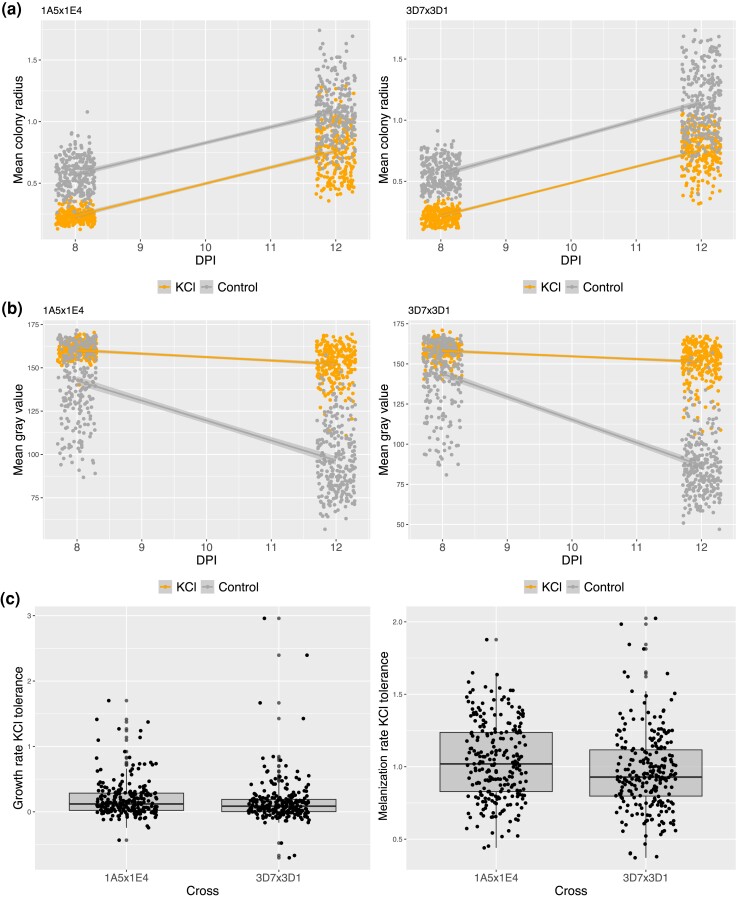
Trait variation in the control and salt stress environments across offspring in each cross. a) Points are mean colony radius in the control and KCl environments at 8 and 12 dpi; smoothed lines demonstrate linear change over time. b) Points are mean gray value in the control and KCL environments at 8 and 12 dpi; smoothed lines demonstrate linear change over time. c) Growth rate KCl tolerance and melanization rate KCl tolerance (KCl tolerance = rate in KCl/rate in control conditions) between the 2 crosses.

A total of 158,480 and 120,059 colonies from the 1A5 × 1E4 and 3D7 × 3D1 crosses, respectively, were phenotyped (see Supplementary Table 2 for details). Differences in growth and melanization between salt stress and control environments and growth rate KCl tolerance and melanization rate KCl tolerance can be seen in Fig. 1. We used paired Wilcoxon signed rank tests to compare mean trait values between the control and KCl treatments (Supplementary Table 2). In the presence of KCl, the colony radius was smaller at 8 and 12 dpi in both crosses, and the growth rate was reduced in the 3D7 × 3D1 cross, but not in the 1A5 × 1E4 cross, suggesting that isolates from the 3D7 × 3D1 cross were more sensitive to KCl (Fig. 1; Supplementary Table 2). This is consistent with the greater growth rate tolerance in the 1A5 × 1E4 cross compared to the 3D7 × 3D1 cross (*F*_(1,487)_ = 9.19, *P* = 0.002, growth rate KCl tolerance: 1A5 × 1E4 = 1.04, 3D7 × 3D1 = 0.96). In the KCl environment, we observed less melanization at both time points and a similar reduced accumulation of melanin over time (melanization rate) in both crosses (Fig. 1; Supplementary Table 2; *F*_(1,486)_ = 2.85, *P* = 0.09, melanization rate KCl tolerance: 1A5 × 1E4 = 0.196, 3D7 × 3D1 = 0.149).

### Correlations between traits

All pairwise correlations (Pearson, nonparametric) between independent measurements are provided in Supplementary Tables 3 and 4. Excluding traits that are statistically or functionally coupled, which includes correlations between 2 time points of the same trait (e.g. colony radius at 8 and 12 dpi in the control), correlations between a rate and the corresponding time point measurements (e.g. gray value at 12 dpi and melanization rate in the control), or correlations between rates and KCl tolerance measurements, we identified many correlations between independent measurements (Supplementary Tables 3 and 4). Notably, the growth rate in the control environment was correlated with the growth rate in the KCl environment in both crosses (*r*(1A5 × 1E4) = 0.63 and *r*(3D7 × 3D1) = 0.62; Supplementary Fig. 1)—thus, isolates that grow faster in a benign environment also grow faster in the presence of salt stress; i.e. they have a higher intrinsic growth rate. Melanization rates showed a similar trend in 1A5 × 1E4—isolates that had higher melanization rates in the benign environment also had higher melanization rates under KCl stress (Supplementary Table 4, *r* = 0.19), but this was not observed in the 3D7 × 3D1 cross (Supplementary Table 3, *r* = 0.07, Supplementary Fig. 1).

We were particularly interested in the relationship between KCl tolerance and melanization, as melanin can help protect cells from osmotic stress. In both crosses, the mean gray value in the control environment at 12 dpi was negatively correlated with growth rate KCl tolerance (Supplementary Tables 2 and 4, 1A5 × 1E4 *r* = −0.26 and 3D7 × 3D1 *r* = −0.43, Supplementary Fig. 2). The relationship at 8 dpi was also negative in both crosses, but not significant in the 3D7 × 3D1 cross (Supplementary Tables 3 and 4, 1A5 × 1E4 *r* = −0.44 and 3D7 × 31 *r* = −0.12, Supplementary Fig. 2). As lower mean gray values correspond to higher amounts of melanin, these correlations indicate that isolates that produce more melanin in a benign environment have a growth rate that is more tolerant to KCl stress, although this relationship was not significant at 8 dpi in the 3D7 × 3D1 cross.

### QTL results

**Fig. 2.**
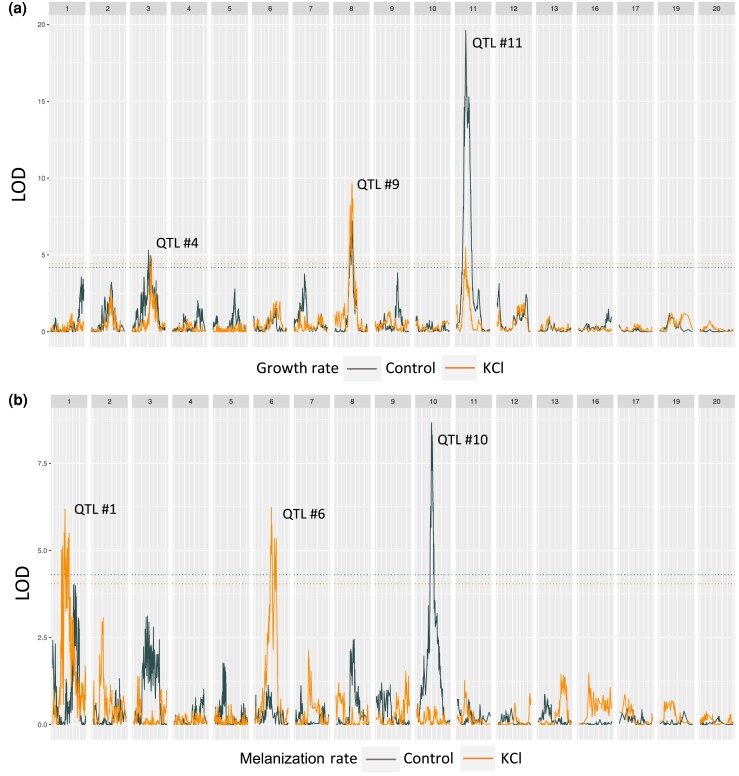
LOD plots from interval mapping for representative traits a) growth rate and b) melanization rate in the control environment (gray) and in the KCl environment (orange) in the 3D7 × 3D1 cross. Each subpanel is a chromosome with the chromosome number indicated at the top. Some numbers are missing because *Z. tritici* carries 8 accessory chromosomes (Chr 14–21) that show presence/absence polymorphisms among isolates. The dashed horizontal lines indicate the LOD significance thresholds for each trait based on permutation. The numbers above the QTL peaks correspond to the QTL numbers from Table 2. For LOD plots of all traits, see Supplementary Fig. 3.

**Fig. 3.**
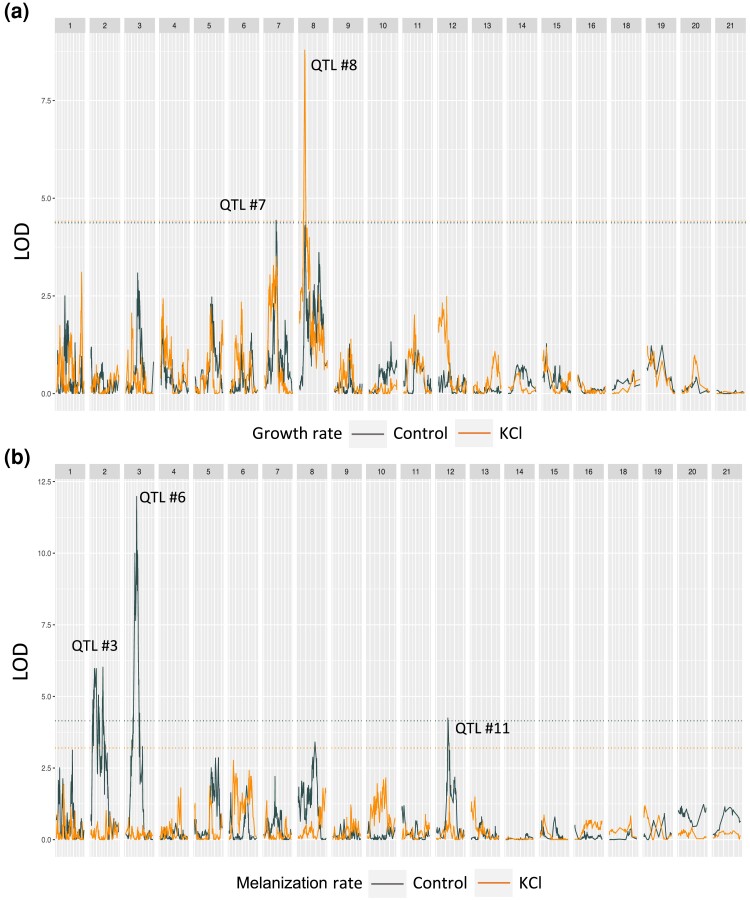
LOD plots from interval mapping for representative traits a) growth rate and b) melanization rate in the control environment (gray) and in the KCl environment (orange) in the 1A5 × 1E4 cross. Some numbers are missing because *Z. tritici* carries 8 accessory chromosomes (Chr 14–21) that show presence/absence polymorphisms among isolates. The dashed horizontal lines indicate the LOD significance thresholds for each trait based on permutation. The numbers above the QTL peaks correspond to the QTL numbers from Table 3. For LOD plots of all traits, see Supplementary Fig. 4.

We identified at least 1 significant QTL for all traits in the 3D7 × 2D1 cross and all but 4 traits (melanization rate and mean gray value at 12 dpi in the KCl environment, melanization rate KCl tolerance at 12 dpi, and melanization rate KCl tolerance) in the 1A5 × 1E4 cross (Table 1). In general, the traits lacking significant QTL had relatively low heritability (Table 1), and thus the power to detect a QTL for those traits was relatively low. All QTL positions, interval sizes, and numbers of genes within the interval are presented in Tables 2 and 3. Largely overlapping QTLs were grouped and given a unique QTL identifier. Examples of how traits measured in different environments can have overlapping QTL intervals are shown in Fig. 2. The QTL no. 4, 9, and 11 are regions of the genome that explain variation in growth rate in both the KCl and control environments. As expected, traits that were highly correlated and/or statistically coupled often had a QTL peak at the same or overlapping positions (e.g. the 1A5 × 1E4 gray values in the control environment at 8 and 12 dpi mapped to the same position on Chr 2: 1,438,934 bp; the 3D7 × 3D1 QTL for colony radius at 12 dpi and growth rate in the control environment mapped to Chr 8: 1,100,862 bp). Logarithm of the odds ratio (LOD) plots for all traits listed in Tables 2 and 3 are provided in the Supplementary Figs. 3 and 4. In Figs. 2 and 3, we provide example plots for only 4 traits (growth rate in KCl, growth rate in the control, melanization rate in KCl, and melanization rate in the control) for the 2 crosses.

**Table 2.** Description of all QTL identified from the 3D7 × 3D1 cross, including map and physical position of the LOD peak marker, the Bayes credible interval length, and the number of genes within the interval.

Type	Chr	Pos (cM)	LOD	Pos (bp)	No. markers	Interval start (bp)	Interval end (bp)	Interval size (bp)	No. genes	PVE	QTL number
Melanization rate in KCl	1	457.43	6.18	1,590,235	2,915	1,035,571	2,769,729	1,734.158	598	0.10	1^M^
Melanization rate KCl tolerance	1	540.00	3.40	2,295,356	9,000	788,430	6,389,989	5,601.559	1,977	0.06	1^M^
Gray value in KCl at 12 dpi	1	576.00	5.99	2,717,234	519	2,295,128	2,769,729	474.601	174	0.10	1^M^
Gray value in KCl at 12 dpi	2	261.00	5.07	691,536	141	679,366	711,981	32.615	15	0.09	2^M^
Colony radius KCl tolerance at 12 dpi	3	142.92	4.49	719,549	553	396,401	944,712	548.311	125	0.08	3^S^
Colony radius KCl tolerance at 8 dpi	3	144.00	14.62	737,773	105	588,483	751,983	163.5	36	0.23	3^S^
Colony radius in KCl at 8 dpi	3	144.15	22.83	737,773	94	588,483	737,773	149.29	31	0.33	3^S^
Growth rate in control	3	415.00	5.31	2,537,296	2,204	1,982,744	3,347,765	1,365.021	462	0.09	4
Colony radius in control at 12 dpi	3	456.63	8.11	2,993,469	427	2,971,359	3,196,103	224.744	79	0.13	4
Colony radius in control at 8 dpi	3	482.67	7.09	3,056,536	428	2,982,086	3,199,430	217.344	76	0.12	4
Growth rate in KCl	3	485.65	4.93	3,110,053	845	2,971,359	3,347,765	376.406	132	0.08	4
Colony radius in KCl at 12 dpi	3	485.65	4.99	3,110,053	3,084	516,017	3,203,947	2,687.93	813	0.08	4
Colony radius KCl tolerance at 8 dpi	4	400.00	4.28	1,936,846	1,000	1,845,763	2,448,166	602.403	200	0.07	5^S^
Colony radius in KCl at 8 dpi	4	403.76	4.37	2,013,908	986	1,867,509	2,448,166	580.657	194	0.07	5^S^
Melanization rate in KCl	6	220.14	6.24	1,767,543	479	1,568,163	1,981,193	413.03	124	0.10	6^M^
Colony radius in control at 12 dpi	7	186.78	4.47	1,109,088	1,366	511,101	1,377,451	866.35	265	0.08	7
Gray value in KCl at 8 dpi	8	680.87	5.69	943,669	732	502,305	974,947	472.642	123	0.10	8^M^
Growth rate in control	8	746.72	7.31	1,100,862	542	1,071,962	1,221,280	149.318	65	0.12	9
Colony radius in control at 12 dpi	8	746.72	10.77	1,100,862	274	1,091,579	1,164,224	72.645	29	0.17	9
Growth rate in KCl	8	747.72	9.66	1,100,862	667	1,091,692	1,284,370	192.678	77	0.16	9
Colony radius in KCl at 12 dpi	8	751.61	9.79	1,112,633	670	1,100,862	1,292,397	191.535	76	0.16	9
Colony radius in control at 8 dpi	8	765.72	8.04	1,221,337	751	1,091,475	1,354,349	262.874	108	0.13	9
Gray value KCl tolerance at 8 dpi	8	776.11	4.22	1,350,673	2,608	962,119	2,165,894	1,203.775	457	0.07	9
Gray value in control at 8 dpi	8	776.11	4.35	1,350,706	2,549	981,398	2,175,005	1,193.607	450	0.07	9
Gray value in control at 8 dpi	10	199.98	12.37	775,475	160	769,669	861,699	92.03	32	0.20	10
Gray value KCl tolerance at 8 dpi	10	199.98	10.68	775,475	281	769,669	919,978	150.309	56	0.17	10
Melanization rate in KCl	10	200.35	8.66	792,011	299	769,669	924,425	154.756	59	0.14	10
Colony radius in control at 8 dpi	10	200.72	12.32	792,272	299	769,669	924,425	154.756	59	0.19	10
Colony radius KCl tolerance at 8 dpi	10	211.51	10.58	924,412	283	792,019	924,425	132.406	53	0.17	10
Colony radius KCl tolerance at 12 dpi	10	211.51	6.68	924,414	1,081	749,521	1,302,645	553.124	211	0.11	10
Colony radius in control at 12 dpi	10	212.61	4.92	924,425	540	749,521	1,054,575	305.054	116	0.08	10
Colony radius in control at 8 dpi	11	183.07	4.20	507,361	528	311,631	698,718	387.087	128	0.07	11
Colony radius KCl tolerance at 12 dpi	11	188.28	5.60	581,439	1,340	74,200	890,544	816.344	268	0.09	11
Gray value in KCl at 12 dpi	11	192.28	6.87	605,810	408	463,714	776,764	313.05	99	0.11	11
Growth rate in control	11	193.00	19.63	616,039	5	616,039	658,119	42.08	13	0.29	11
Growth rate in KCl	11	193.00	5.47	616,039	219	468,760	698,718	229.958	72	0.09	11
Colony radius in control at 12 dpi	11	193.00	16.79	616,039	25	601,600	658,119	56.519	16	0.26	11
Gray value in control at 12 dpi	11	194.11	6.64	657,530	498	514,049	870,059	356.01	121	0.11	11
Gray value KCl tolerance at 12 dpi	11	194.11	5.55	657,530	700	367,519	870,059	502.54	168	0.09	11
Gray value in control at 8 dpi	11	194.11	5.30	658,070	244	463,714	704,331	240.617	77	0.09	11
Gray value in KCl at 8 dpi	11	194.11	17.09	658,119	5	616,039	658,119	42.08	13	0.26	11
Growth rate KCl tolerance	11	195.28	7.45	658,119	370	500,942	802,650	301.708	98	0.12	11

PVE is the proportion of variance explained by the QTL. QTL number is a number assigned to a particular QTL peak that groups overlapping QTLs. Superscript letters indicate QTL unique to KCL environment for size-related (^S^) and melanin-related traits (^M^).

**Table 3.** Description of all QTLs identified from the 1A5 × 1E4 cross, including map and physical position of the LOD peak marker, the Bayes credible interval length, and the number of genes within the interval.

Type	Chr	Pos (cM)	LOD	Pos (bp)	No. markers	Interval start (bp)	Interval end (bp)	Interval size (bp)	No. genes	PVE	QTL number
Gray value in control at 8 dpi	1	328.91	5.69	572,065	402	442,697	727,696	284.999	92	0.10	1
Gray value KCl tolerance at 8 dpi	1	328.91	5.01	572,065	405	442,697	732,023	289.326	93	0.08	1
Melanization rate KCl tolerance	1	328.91	4.46	572,065	463	461,577	779,936	318.359	102	0.08	1
Colony radius KCl tolerance at 12 dpi	1	559.12	4.07	1,849,200	4,654	399,579	5,725,682	5,326.103	1,964	0.07	2^S^
Growth rate KCl tolerance	1	797.00	4.80	3,405,315	3,068	399,544	3,488,556	3,089.012	1,151	0.08	2^S^
Colony radius in KCl at 12 dpi	1	1104.00	4.03	5,792,644	4,774	372,886	5,956,797	5,583.911	2,056	0.07	2^S^
Melanization rate in control	2	70.00	6.56	574,965	1,211	405,115	1,914,465	1,509.35	509	0.11	3
Gray value in control at 12 dpi	2	204.66	4.55	1,438,934	618	1,284,536	2,152,463	867.927	281	0.08	4
Gray value in control at 8 dpi	2	204.66	18.08	1,438,934	185	1,438,863	1,748,215	309.352	95	0.27	4
Gray value KCl tolerance at 8 dpi	2	204.66	15.97	1,438,934	184	1,438,934	1,748,215	309.281	95	0.24	4
Colony radius in control at 8 dpi	2	211.42	8.76	1,631,840	217	1,438,934	1,776,132	337.198	105	0.14	4
Gray value KCl tolerance at 8 dpi	3	147.00	6.15	1,325,440	210	1,243,932	1,684,531	440.599	179	0.10	5
Gray value in control at 8 dpi	3	148.00	6.66	1,325,440	133	1,325,422	1,592,801	267.379	115	0.11	5
Colony radius in control at 8 dpi	3	163.74	5.83	1,521,535	725	355,042	1,592,801	1,237.759	374	0.10	5
Melanization rate in control	3	193.38	11.99	1,711,037	290	1,489,966	2,105,601	615.635	180	0.19	6
Gray value in control at 12 dpi	3	198.73	4.04	1,928,783	860	1,743,111	2,927,356	1,184.245	361	0.07	6
Growth rate KCl tolerance	5	399.00	4.34	2,638,913	155	2,511,016	2,658,480	147.464	48	0.07	7^S^
Colony radius in KCl at 8 dpi	6	179.00	4.48	1,512,508	594	778,583	1,557,528	778.945	256	0.08	8^S^
Growth rate in control	7	158.82	4.43	539,824	255	437,699	651,318	213.619	80	0.07	9
Colony radius in control at 12 dpi	7	158.82	4.67	539,837	254	437,718	651,318	213.6	80	0.08	9
Growth rate in KCl	8	76.80	8.79	274,792	82	256,529	346,224	89.695	25	0.14	10
Colony radius in KCl at 12 dpi	8	76.80	8.94	274,792	67	256,529	313,758	57.229	17	0.15	10
Gray value in control at 12 dpi	8	78.58	4.92	302,772	882	89,684	921,404	831.72	308	0.08	10
Gray value in control at 8 dpi	8	79.00	12.12	302,772	66	270,834	346,224	75.39	22	0.19	10
Colony radius in control at 12 dpi	8	79.00	9.72	302,772	80	260,355	346,224	85.869	24	0.16	10
Colony radius in control at 8 dpi	8	79.00	11.06	302,772	415	260,101	732,444	472.343	163	0.18	10
Gray value KCl tolerance at 8 dpi	8	79.00	8.26	302,772	339	89,573	346,224	256.651	90	0.14	10
Colony radius KCl tolerance at 8 dpi	8	137.00	5.05	832,180	1,177	589,084	1,648,406	1,059.322	396	0.08	10
Gray value in KCl at 8 dpi	8	164.00	4.33	1,377,926	1,406	305,075	1,623,437	1,318.362	485	0.07	10
Melanization rate in control	12	126.74	4.24	473,688	275	441,706	821,603	379.897	101	0.07	11

PVE is the proportion of variance explained by the QTL. QTL number is a number assigned to a particular QTL peak that identifies overlapping QTLs. Superscript letters indicate QTL unique to KCL environment for size-related (^S^) and melanin-related traits (^M^).

### QTL shared across environments, traits, and time points

Several QTLs were shared among benign and salt stress environments, across time points and traits. Only one of these shared QTL is novel to this study; the others were found in earlier studies investigating different stressors. The single novel QTL shared between benign and salt stress environments was found in the 3D7 × 3D1 cross on Chr 8 (1,100.86–1,221.33 kb, QTL no. 9, Fig. 2) and explained variation in 7 traits including growth in the control and KCl environments, as well as mean gray value in the control environment and gray value KCl tolerance at 8 dpi (Fig. 2; Table 2).

Of the QTL shared across benign and KCl environments that were identified here and found in earlier studies using different stresses (e.g. cold and fungicide), the most striking, in terms of the number of traits and the magnitude of the LODs, was the QTL on Chr 11 between 507.36 and 658.11 kb (QTL no. 12) in the 3D7 × 3D1 cross. This QTL explained variation in 11 of the 18 traits and in both environments (Table 2). This QTL was identified previously and was found to explain variation in growth, fungicide sensitivity, and melanization in this cross (Lendenmann *et al.* 2014, 2015). The next largest, in terms of number of traits, was a QTL in the 1A5 × 1E4 cross on Chr 8 between 89.573 and 346.224 kb (QTL no. 10) that explained variation in growth rate, colony radius, and gray value in both control and KCl environments (Fig. 3; Table 3). This QTL on Chr 8 was also found to explain variation in response to oxidative stress (Zhong *et al.* 2021). Another notable QTL in terms of high LOD (>10) was on Chr 10 (749.521–1,302.645 kb, QTL no. 10) in the 3D7 × 3D1 cross, which explained variation in melanization rate and colony radius in the control environment (Table 2). This QTL was previously found to explain variation in growth-related traits at 10°C (Lendenmann *et al.* 2014) and under oxidative stress (Zhong *et al.* 2021). This QTL contains the ZT3D7 ortholog (ZT3D7_G9993) of the HOG1 gene annotated in the IPO323 genome (MgHOG1 Mycgr3G76502).

### Unique QTL associated with the KCl environment and pathway enrichment analysis

Several novel QTLs that were unique to the KCl environment were also found (indicated with an ^S or M^ in Table 2 and 3). In the 3D7 × 3D1 cross, 2 QTLs were found for traits related to colony radius: the first on Chr 3 (QTL no. 3) explained variation in colony radius in KCl at 8 dpi and colony radius KCl tolerance at 8 and 12 dpi; and the second on Chr 4 (QTL no. 5) explained variation in colony radius in KCl at 8 dpi and colony radius KCl tolerance at 8 dpi. For melanin-related traits, 4 QTLs were found in the 3D7 × 3D1 cross: the first on Chr 1 (QTL no. 1) explained variation in 3 traits (melanization rate in KCl, melanization rate KCl tolerance, and gray value in KCL at 12 dpi); the second on Chr 2 (QTL no. 2) explained variation in gray value in KCl at 12 dpi; the third on Chr 6 (QTL no. 6) explained variation in melanization rate in KCl; and the fourth on Chr 8 (QTL no. 8) explained variation in gray value in KCl at 8 dpi. In the 1A5 × 1E4 cross, a total of 3 QTLs related to colony radius in KCl were found. The first was on Chr 1: QTL no. 2 explained variation in colony radius KCl tolerance at 12 dpi, growth rate KCl tolerance, and colony radius in KCl at 12 dpi. The second unique KCl environment QTL was on Chr 5 (QTL no. 7) and explained variation in growth rate KCl tolerance; the fifth on Chr 6 (QTL no. 8) explained variation in radius at 8 dpi in KCl. No unique QTLs were found for melanin-related traits in the 1A5 × 1E4 cross.

For pathway enrichment analyses, we focused on traits that had significant QTLs unique to the KCl environment (indicated in Tables 2 and 3 with ^S or M^) and investigated if genes associated with those traits were enriched for KEGG pathways or GO annotations. We analyzed each trait-QTL separately and did the same analysis using all genes for size- or melanin-related traits combined (Supplementary Tables 5 and 6). No significantly enriched KEGG pathways were identified in our data. For the GO enrichment, we report on the results for size- or melanin-related trait-QTL combined, and we use the raw *P*-values instead of the adjusted *P*-values as indicators of possible GO enrichment because adjusting *P*-values in GO enrichment analysis may be too conservative and inappropriate (Alexa and Rahnenfuhrer 2023). All results are presented in Supplementary Tables 5 and 6. Considering all genes within size-related unique KCl QTL combined in the 3D7 × 3D1 cross (Table 2, QTL no. ^S^): for BP double-strand break repair via nonhomologous end joining (GO: 0006303) and nucleotide-excision repair (GO: 0006289), GO terms were enriched; for CC Ku70:Ku80 complex (GO: 0043564), GO term was enriched; and for MF GTPase activity (GO: 0003924), superoxide dismutase activity (GO: 0004784), telomeric DNA binding (GO: 0042162), and damaged DNA-binding (GO: 0003684), GO terms were enriched (Supplementary Table 5b). There were 15 genes within these QTLs that have 1 or more of these GO annotations (Supplementary Table 7a). For genes within melanin-related unique KCl QTL combined in the 3D7 × 3D1 cross (Table 2, QTL no. ^M^): for BP glutathione biosynthetic process (GO: 0006750), GO terms were enriched; for CC, no GO term was enriched; and for MF chaperone binding (GO: 0051087), phosphatidylinositol phospholipase C activity (GO: 0004435), and shikimate 3-dehydrogenase (NADP+) activity (GO: 0004764), GO terms were enriched (Supplementary Table 5d). There were 13 genes within these QTLs that have 1 or more of these GO annotations (Supplementary Table 7b). Considering all genes within size-related unique KCl QTL combined in the 1A5 × 1E4 cross (Table 2, QTL no. ^S^): for BP, no GO terms were enriched; for CC cell wall (GO: 0005618), GO term was enriched; and for MF chaperone binding (GO: 0051087) and triglyceride lipase activity (GO: 0004806), GO terms were enriched (Supplementary Table 6b). There were 12 genes within these QTLs that have 1 or more of these GO annotations (Supplementary Table 7c). There were no unique KCl melanin-related QTL in the 1A5 × 1E4 cross.

### Analysis of QTL no. 3 in 3D7 × 3D1 and identification of candidate genes

**Fig. 4.**
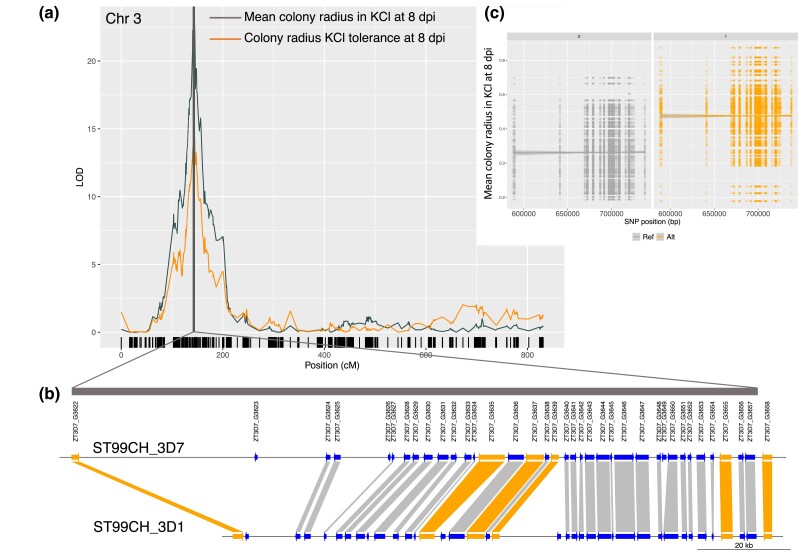
QTL on chromosome 3 for 3 traits related to growth under osmotic stress in the 3D7 × 3D1 cross. The 3 traits were colony radius at 8 dpi and colony radius KCl tolerance at 8 and 12 dpi (KCl tolerance = colony radius in KCl/colony radius in control conditions). a) LOD peak and Bayes credible interval (shaded) from interval mapping, with SNP positions indicated on the *x*-axis as black vertical lines. b) Alignment of the 36 genes within the QTL interval between the 3D7 and 3D1 parental strains; orange-colored genes are the 6 most promising candidate genes described in the main text and c) allelic effects of 94 SNPs within the QTL interval on mean colony radius in KCl at 8 dpi. Isolates carrying the reference allele (ref = 3D7) shown in gray have smaller colonies compared to the alternative allele (alt = 3D1) shown in orange. SNP position in base pairs is shown on the *x*-axis. The marker with the highest LOD is at position 737,773 bp.

To identify candidate genes associated with KCl stress, we focused attention on novel QTL with a high LOD (>10), relatively narrow intervals, and that explained a relatively high proportion of the variation for a KCl-associated trait. Only QTL no. 3 in the 3D7 × 3D1 cross met these requirements (Table 2). This QTL had a high LOD (>10) for colony radius at 8 dpi in the KCl environment and colony radius KCl tolerance at 8 dpi. The LOD peaked at 22.8 at chromosome position 737,773 bp. This QTL explained 33% of the phenotypic variance in colony radius at 8 dpi and 23% of the variance in colony radius KCl tolerance at 8 dpi. Isolates with the nonreference allele at the QTL peak marker had larger colonies at 8 dpi (*F* = 113.1, *df* = 1.233, *P* ≤ 0.001, Fig. 4c) and greater colony radius KCl tolerance at 8 dpi (*F* = 56.07, *df* = 1.23, *P* ≤ 0.001). The Bayes confidence interval for the QTL for colony radius in KCl at 8 dpi spanned 149.29 kb and contained 94 SNPs and 36 genes (Table 2; Fig. 4b). For this interval, we present data on the genes in the reference genome (3D7), the orthologous genes in 3D1, the gene sequence similarity (including ±200 bp of flanking regions) between the parents, the gene's putative encoded function (PFAM database), and if there is any evidence of differential gene expression between the parents across all available data sets (Table 4; see Supplementary Table 8 for full details).

**Table 4.** Genes within the QTL interval for colony radius at 8 dpi in KCl and colony radius KCl tolerance at 8 dpi in the 3D7 × 3D1 cross.

Chr	Gene id 3D7	Gene start	Gene end	Gene id 3D1	Sequence similarity (%)	DGE	Annotation
**3**	**ZT3D7_G3622**	**598091**	**599743**	**ZT3D1_G3567**	**97**.**68**	**Yes**	**Fungal-specific transcription factor domain**
3	ZT3D7_G3623	637954	638542	ZT3D1_G7387	96.23	NA	
3	ZT3D7_G3624	653468	654397	ZT3D1_G3569	90.78	Yes	Protein of unknown function (DUF1349)
3	ZT3D7_G3625	655237	656628	ZT3D1_G3570	97.04	Yes	
3	ZT3D7_G3626	667030	667567	NA		Yes	Pterin 4 alpha carbinolamine dehydratase
3	ZT3D7_G3627	667812	668321	ZT3D1_G3571	98.46	Yes	
3	ZT3D7_G3628	670445	671787	ZT3D1_G3572	100.00	Yes	Transketolase, pyrimidine binding domain, transketolase, C-terminal domain
3	ZT3D7_G3629	672336	673658	ZT3D1_G3573	99.83	NA	
3	ZT3D7_G3630	674777	676658	ZT3D1_G3574	99.96	Yes	Dehydratase family
3	ZT3D7_G3631	677782	680135	ZT3D1_G3576	97.72	Yes	ABC transporter, ABC transporter
3	ZT3D7_G3632	680734	681942	ZT3D1_G3577	97.73	Yes	Epoxide hydrolase N terminus
3	ZT3D7_G3633	683647	685131	ZT3D1_G3578	81.12	YA	Phosphotransferase enzyme family
3	ZT3D7_G3634	685575	685937	ZT3D1_G3579	98.96	Yes	
**3**	**ZT3D7_G3635**	**686721**	**692363**	**ZT3D1_G3580**	**99**.**07**	**Yes**	**Protein kinase domain, protein kinase C-terminal domain, N-terminal half of MaoC dehydratase, MaoC-like domain, C2 domain**
3	ZT3D7_G3636	693114	696535	ZT3D1_G3582	99.50	Yes	Importin-beta N-terminal domain, Cse1
**3**	**ZT3D7_G3637**	**697014**	**700871**	**ZT3D1_G3583**	**97**.**44**	**Yes**	**DEAD/DEAH box helicase, rRNA-processing arch domain, DSHCT (NUC185) domain, Ski2 N-terminal region, helicase conserved C-terminal domain**
3	ZT3D7_G3638	701182	702047	ZT3D1_G3584	98.74	Yes	short chain dehydrogenase
**3**	**ZT3D7_G3639**	**702431**	**704143**	**ZT3D1_G3585**	**97**.**16**	**Yes**	**Major facilitator superfamily**
3	ZT3D7_G3640	705425	706303	ZT3D1_G3587	99.91	Yes	
3	ZT3D7_G3641	706740	707975	ZT3D1_G3588	98.53	Yes	
3	ZT3D7_G3642	708779	709336	ZT3D1_G3589	98.33	NA	
3	ZT3D7_G3643	709874	711886	ZT3D1_G3590	94.46	Yes	Ubiquitin-conjugating enzyme
3	ZT3D7_G3644	712327	715212	ZT3D1_G3591	98.93	Yes	Utp14 protein
3	ZT3D7_G3645	715430	715798	ZT3D1_G3592	99.74	No	Mitochondrial ribosomal protein L27
3	ZT3D7_G3646	716302	720497	ZT3D1_G3593	98.96	NA	Staphylococcal nuclease homolog, short chain dehydrogenase, enoyl-(acyl carrier protein) reductase, Tudor domain
3	ZT3D7_G3647	720900	723737	ZT3D1_G3594	99.38	Yes	PQQ-like domain, ER membrane protein complex subunit 1, C-terminal
3	ZT3D7_G3648	725571	726437	ZT3D1_G3595	99.02	No	Ras family
3	ZT3D7_G3649	726651	727599	NA		Yes	NmrA-like family
3	ZT3D7_G3650	727935	729979	ZT3D1_G3596	98.53	No	Heterokaryon incompatibility protein (HET)
3	ZT3D7_G3651	730698	731819	ZT3D1_G3597	99.93	Yes	Transaldolase/fructose-6-phosphate aldolase
3	ZT3D7_G3652	732329	733083	ZT3D1_G3598	96.53	Yes	Copper/zinc superoxide dismutase (SODC)
3	ZT3D7_G3653	734230	736078	ZT3D1_G3599	99.73	Yes	FGGY family of carbohydrate kinases, C-terminal domain, FGGY family of carbohydrate kinases, N-terminal domain
3	ZT3D7_G3654	737260	737987	ZT3D1_G3600	99.87	Yes	
**3**	**ZT3D7_G3655**	**739211**	**741757**	**ZT3D1_G3601**	**99**.**97**	**Yes**	**CBS domain, voltage-gated chloride channel**
3	ZT3D7_G3656	743352	744442	ZT3D1_G3602	99.73	Yes	Protein of unknown function (DUF1295)
3	ZT3D7_G3657	744744	746896	ZT3D1_G3603	99.92	Yes	Pro-kumamolisin, activation domain
**3**	**ZT3D7_G3658**	**748505**	**750639**	**ZT3D1_G3604**	**100**	**Yes**	**PX domain, Vps5 C-terminal like**

Bold indicates genes that are the most promising candidate genes, as described in the main text. Gene sequence similarity between the 2 parental strains was obtained using BLAST.Differential gene expression (DGE) column indicates if the gene was differentially expressed between parents in any one of the data sets used (in planta or in vitro, see Methods in main text). The annotations were obtained using InterProScan using multiple protein databases; here, we show only the PFAM annotation.

Several genes had SNP variation predicted to have a high impact based on SNPEff analysis (Supplementary Table 9, *ZT3D7_G3622*, *ZT3D7_G3623*, *ZT3D7_G3624*, *ZT3D7_G3632*, *ZT3D7_G3633*, *ZT3D7_G3635*, and *ZT3D7_G3649*). A transposable element (6,927 bp) was found within 200 bp of *ZT3D7_G3623*, and 1 gene, *ZT3D7_G3649*, is a singleton, with no ortholog present in any of the other 18 *Z. tritici* reference genome sequences (Supplementary Table 8). Two genes within this QTL interval and another gene immediately adjacent to the interval have GO annotations that were enriched (ZT3D7_G3648, ZT3D7_G3652, and ZT3D7_G3659, Supplementary Table 7). ZT3D7_G3648 and ZT3D7_G3659 have predicted GTPase activity (GO: 0003924), and ZT3D7_G3652 has predicted superoxide dismutase activity (GO: 0004784). The SNP variation in these genes between the parents was not predicted to have a large impact on the protein (Supplementary Table 9). Based on predicted protein function, and sequence and gene expression variation between the parents, we consider 6 genes to be the most promising candidates for further analyses: *ZT3D7_G3622*, *ZT3D7_G3635*, *ZT3D7_G3637*, *ZT3D7_G3639*, *ZT3D7_G3655*, and *ZT3D7_G3658* (this order reflects their relative positions on chromosome 3).

*ZT3D7_G3622* has a loss of function SNP (Chr 3: 598092) in 3D1 that is predicted to cause a loss of a predicted start codon. The gene contains a single exon, encodes a protein length of 550 amino acids (aa), and is predicted to contain a fungal-specific TF domain and Zn(2)-C6 fungal-type DNA-binding domain (Table 4). The ortholog in the 3D1 genome is *ZT3D1_G3567*. It encodes a longer protein (722 aa) and has 2 exons: the first exon's product encodes a Zn(2)-C6 fungal-type DNA-binding domain, and the second exon's product is the same as that of *ZT3D7_G3622*. The predicted protein in 3D1 is the same as the ortholog in IPO323 (*Mycgr3T108446*). Although we do have reads mapping to this region, and when grown in vitro on MM this gene is downregulated in 3D1 compared to 3D7 (Supplementary Table 8), our RNA sequence data do not clearly support gene models in either 3D1 or 3D7, there are no clear exon/intron boundaries. This suggests that the annotation of this gene may require manual curation.

*ZT3D7_G3635* has a high-impact SNP variant (Chr 3: 681906) that is predicted to cause a frameshift. The gene has 6 exons in 3D7 and encodes a protein length of 1,078 aa. Based on INTERPROSCAN and Ensemble Fungi, the protein has multiple predicted domains; the largest is a protein kinase domain. The protein also contains a serine/threonine-protein kinase active site, and the MF of the first GO term (GO: 0004674) is protein serine/threonine kinase activity, suggesting that this gene is likely to be involved in osmotic stress response (Hohmann 2002). The RNA sequence data support the gene model. The ortholog in 3D1 is *ZT3D1_G3580*, which has 3 exons and multiple predicted domains that do not differ from those predicted for *ZT3D7_G3635*. The gene is upregulated in vitro on YSB and downregulated in planta at 28d in 3D1 compared to 3D7 (Supplementary Table 8).

*ZT3D7_G3637* has 1 exon, encodes a protein length of 1,285 aa, and includes multiple predicted domains including a DEAD/DEAH box helicase, which is involved in plant responses to osmotic stress (Nguyen *et al.* 2018). The ortholog in 3D1 (*ZT3D1_G3583*) encodes a similar protein as *ZT3D7_G3637*, has the same length, and has the same predicted domains. There are no high-impact SNPs between the parents, but several moderate and low-impact variants were identified (Supplementary Table 9). The RNA sequence data support the annotation, and the gene is downregulated in 3D1 in both YSB and MM (Supplementary Table 8).

*ZT3D7_G3639* has 4 exons in 3D7. The gene sequence similarity between 3D7 and 3D1 is 98.66%, and there is an 8-bp intronic deletion in 3D1. This gene belongs to the major facilitator superfamily (MFS) and encodes a protein length of 512 aa. According to the Phobius analysis, the gene contains 12 transmembrane segments (Supplementary Table 8). Proteins with transmembrane activity and transmembrane segments are likely to play an important role in sensing and regulating osmotic stress (Hohmann 2002). The ortholog in 3D1 is *ZT3D1_G3580*, which has the same number of exons, encodes the same protein length, and has the same predicted function. The RNA sequence data support the gene model, and the differential expression analysis found that the gene is downregulated in planta at 12 and 14 days in 3D1 compared to 3D7 (Supplementary Table 8).

*ZT3D7_G3655* has 1 exon and encodes a protein length of 848 aa. The gene contains 10 transmembrane segments (Supplementary Table 8) and encodes a voltage-gated chloride channel domain; thus, it is likely to be involved in osmotic stress responses. The gene sequence between the parents is almost identical, but there is 1 moderate effect SNP within the gene (Supplementary Table 9). When grown in vitro on MM, this gene is upregulated in 3D1 compared to 3D7. The RNA sequence data support the gene model annotation.

*ZT3D7_G3658* has 4 exons and encodes a protein length of 614 aa. Its ortholog in 3D1 (*ZT3D1_G3604*) has 3 exons and encodes 572 aa. The gene encodes a vacuolar protein sorting-associated protein 17, Vps17. Vacuoles play an important role in osmotic stress responses: their fission and fusion enable cells to maintain turgor pressure and regulate cell volume (Richards *et al.* 2010). Comparison of the 3D1 gene sequence to the 3D7 genome using BLAST revealed that the 3D7 gene sequence was identical, but the 3D1 ortholog annotation is shorter and has only 3 exons. Our RNA sequence data support the 3D1 annotation with only 3 exons, as there were no RNA sequence reads mapping to exon 1 of the *ZT3D7_G36580*. This gene was downregulated in vitro in both MM and YSB in 3D1 compared to 3D7.

## Discussion

We were able to map major effect loci affecting colony growth and melanization in the presence of KCl in both crosses. We focused on QTLs that were unique to the KCl environment to identify candidate genes that are specific to osmotic stress, as opposed to finding genes that may be more generally related to intrinsic growth or melanin production. A QTL on Chr 3 in the 3D7 × 3D1 cross was the most promising for identifying genes specifically associated with KCl stress. We highlighted the 6 most promising candidate genes in this QTL based on their predicted encoded functions, sequence similarity, and transcriptional profiles. We also identified several QTLs shared across environments and confirmed that several previously identified QTLs, which explained variation in growth and melanization across different environmental stressors (Table 2 and 3; e.g. 3D7 × 3D1 Chr 11 and 1A5 × 1E4 Chr 8) and also explained trait variation in the presence of osmotic stress.

### Effect of KCl on colonies

In the presence of KCl at 0.75 M, colonies were typically smaller and less melanized compared to the control environment (Fig. 1; Supplementary Table 2). A reduction in colony radius in the KCl environment supports our hypothesis that this environment was stressful to *Z. tritici*. Our results suggest that the growth rate in the 1A5 × 1E4 cross was less affected by KCl-induced osmotic stress, as the mean growth rate was not reduced in the KCl environment, compared to the 3D7 × 3D1 cross that exhibited a significantly reduced growth rate (Supplementary Table 2). However, we note that the overall difference in growth rate in the 3D7 × 3D1 progeny between the control and KCl environments was relatively small (∼6% reduction in growth; Supplementary Table 2).

When experiencing osmotic stress, an increase in melanin is thought to protect cells by strengthening the fungal cell wall, but a reduced metabolic capacity under stress may limit the ability of a cell to produce melanin. The mean colony gray values were higher, indicative of lower levels of melanin, for strains grown in the KCl environment (Supplementary Table 2). This suggests that the *Z. tritici* response to osmotic stress did not involve an increased production of melanin to protect the cells as was observed in halotolerant species (Kogej *et al.* 2007). We did observe, however, that isolates that were more melanized in the benign control environment (lower gray value) were more tolerant to salt stress (Supplementary Fig. 2); their growth rates were higher, and their reductions in melanization were lower compared to isolates that were less melanized in the control environment (Supplementary Tables 3 and 4). This suggests that strains having a greater intrinsic capacity to become melanized are more tolerant to salt stress.

### Shared QTL

Some QTLs that explained variation in growth and melanization in the stressful environment overlapped with QTL for trait variation in the control environment. Prominent examples include the Chr 11 QTL no. 11 in the 3D7 × 3D1 cross and the Chr 8 QTL no. 10 in the 1A5 × 1E4 cross. These genomic regions may harbor genes that affect the intrinsic growth of colonies under a wide range of environments and/or genes with pleiotropic effects. Many of the QTLs shared across environments were identified in earlier experiments and discussed previously (Lendenmann *et al.* 2015; Zhong *et al.* 2021). The QTL no. 11 in the 3D7 × 3D1 was shown to be due to differential expression of the *Zmr1* gene encoding a TF that regulates the synthesis of DHN melanin in *Z. tritici* (Krishnan *et al.* 2018). *Zmr1* was shown to have pleiotropic effects on melanin production, colony growth rates, and fungicide resistance (Krishnan *et al.* 2018). One shared QTL from the 3D7 × 3D1 cross (QTL no.10) contained the Hog1 ortholog (ZT3D7_G9993). This gene is known to regulate the osmoadaptive response, virulence, and the response to fungicides (Mehrabi *et al.* 2006).

### QTL uniquely associated with the KCl environment

This QTL analysis enabled us to identify large-effect loci that explain variation in traits under KCl stress. Among the QTL uniquely associated with KCl stress, most had moderate LODs with each QTL explaining only a small proportion of the phenotypic variance (3D7 × 3D1 maximum = 0.10, 1A5 × 1E4 maximum = 0.11). The exception was QTL no. 3 in the 3D7 × 3D1 cross, which explained up to 33% of the phenotypic variance. This QTL on chromosome 3 was associated with differences in colony radius at 8 dpi and colony radius KCl tolerance at 8 and 12 dpi. We targeted this QTL for further investigation, seeking to identify genes specifically associated with KCl tolerance. It is interesting to note that no LOD peak was detected for colony radius at 12 dpi, even though colony radius at 12 dpi was correlated with colony radius at 8 dpi (Supplementary Table 4, correlation = 0.58, *P* < 0.001). As the growth rate is based on the change in colony radius between 8 and 12 dpi, we might expect the growth rate in KCl to share the QTL for colony radius at 8 dpi. To better understand why growth rate does not share this QTL, we examined the trait correlations. The growth rate is highly correlated with colony radius at 12 dpi (0.93) and less strongly correlated with colony radius at 8 dpi (0.26) (Supplementary Table 4). There is less variation in colony radius at 8 dpi; thus, colony radius at 12 dpi has a greater impact on the growth rate. The QTL no. 3 also explained variation in colony radius KCl tolerance at 8 and 12 dpi, although the association with this trait at 12 dpi explained relatively little phenotypic variation (0.08). Thus, the QTL peak is more strongly associated with colony radius and colony radius KCl tolerance at an earlier stage of colony growth. A similar pattern was observed in the chromosome 11 QTL associated with melanization (Lendenmann *et al.* 2014; Krishnan *et al.* 2018), with the QTL LOD score very high in the younger colonies and decreasing as colonies aged. After cloning and characterizing the *Zmr1* gene and its functions, this pattern could be explained based on the finding that the difference in *Zmr1* expression (and the resulting difference in melanization) was significantly higher in younger colonies than in older colonies (Krishnan *et al.* 2018). We hypothesize that QTL no. 3 associated with KCl tolerance is underpinned by a similar mechanism that is specific to the earlier stage of colony growth.

### Candidate genes in the QTL for colony radius and colony radius KCl tolerance at 8 dpi in the 3D7 × 3D1 cross

Among the 36 genes found in the interval spanning this QTL no. 3, we consider 6 genes to be the most promising candidates to be involved in sensing and responding to salt stress and responsible for this QTL. Below, we consider each in turn in the order of their relative positions on chromosome 3.

The first candidate gene, *ZT3D7_G3622*, is predicted to encode a fungal-specific TF. The ortholog in the IPO323 reference genome (assembly MYCGR v2.0) is *Mycgr3T108446*, and it has 21 paralogs. TFs regulate gene expression and are important regulators of molecular responses to environmental stimuli (e.g. Hohmann 2002; Shi *et al.* 2021), making this gene a good candidate. The *Zmr1* gene identified in this same cross and shown to be responsible for differences in melanization, with greater effects in younger colonies, is a TF (Krishnan *et al.* 2018). The SNP variant in 3D1 (Chr 3: 598092) is predicted to cause a loss of the start codon that is likely to cause a loss of function. In *F. graminearum*, mutants lacking a similar TF (called *Fss1*) were sensitive to high sodium and lithium, but they were not sensitive to elevated potassium (Son *et al.* 2015).

The second candidate, *ZT3D7_G3635*, is predicted to encode a serine/threonine-protein kinase active site, which is known to regulate MAP kinases (Hohmann 2002; Li *et al.* 2019). A SNP variant found in the 3D1 allele is predicted to cause a frameshift that is likely to cause a loss of function. The ortholog in the IPO323 genome is *Mycgr3G56270*, and it has 1 paralog in the reference genome. A serine/threonine kinase from *Aspergillus flavus*, *AflSte20*, was recently functionally validated using a knockout mutant. This study showed that the knockout had higher sensitivity to osmotic stress compared to the wild type (Li *et al.* 2019), confirming the role of this serine/threonine-protein kinase in osmotic stress response in a different fungal plant pathogen.

The third candidate, *ZT3D7_G3637*, is predicted to encode a DEAD/DEAH box helicase domain. The reference genome ortholog is *Mycgr3G99415*, and it has 6 paralogs. The DEAD-box helicases are the largest RNA helicase (RH) subfamily. In plants, they play multiple functional roles and are important in stress responses (Pandey *et al.* 2020). For example, in *Arabidopsis thaliana*, *AtRH17* is involved in salt stress tolerance (Nguyen *et al.* 2018); in rice, *OsABP* is upregulated in response to salt (Macovei *et al.* 2012); and in barley, HvD1 accumulated in salt- and cold-stressed plants (Nakamura *et al.* 2004). In wheat, the DEAD-box helicase *TaRH1* that was first identified as a component of the defense response to stripe rust (*Puccinia striiformis* f. sp. *tritici*) was also found to be involved in plant response to multiple stressors, including salt stress (Zhang *et al.* 2014). SNP variation found between the parents was predicted to have only moderate effects; no high-impact SNPs were detected. We chose this gene as a promising candidate based mainly on its known associations with salt stress in plants and the significant differences in the expression found between 3D1 and 3D7 while growing in vitro.

A gene encoding a MFS domain, *ZT3D7_G3639*, is the fourth candidate we highlight. The reference genome ortholog is *Mycgr3G85019*, and it has 58 paralogs. MFS transporters facilitate the movement of small solutes across cell membranes in response to chemiosmotic gradients, making them likely to play an important role in sensing and regulating osmotic stress (Hohmann 2002). We chose this gene as a promising candidate based mainly on its known functions and associations with multiple stressors including fungicides (Dos Santos *et al.* 2014; Omrane *et al.* 2015).

The fifth highlighted gene is *ZT3D7_G3655* that encodes a voltage-gated chloride channel domain and membrane transport domains. The reference genome ortholog is *Mycgr3T20761*, and there are 3 paralogs in the IPO323 reference genome. Chloride channels are important in animal cell volume regulation (Jentsch *et al.* 2002), fungal oxidative stress response (Oddon *et al.* 2007), fungal pathogen virulence (Zhu and Williamson 2003; Cañero and Roncero 2008), and plant immunity (Guo *et al.* 2014). The role of chloride channels in response to salt stress is well established in animals (Jentsch *et al.* 2002) and to a lesser degree in plants (Barbier-Brygoo *et al.* 2011), but less is known from fungi. For one of the better-known families of chloride channels, the CLC family, a single gene (*GEF1*) has been identified in yeast. In 2 pathogenic fungi (*Cryptococcus neoformans* and *Fusarium oxysporum*), the GEF1 homolog has been shown to influence virulence (Zhu and Williamson 2003; Cañero and Roncero 2008). The gene sequence between the parents is highly conserved; however, gene expression differences were observed in minimal media, so differences may exist in the regulatory sequence.

The final candidate we highlight is *ZT3D7_G3658*, which encodes a vacuolar-associated protein 17, *Vps17*. The orthologous gene in the reference genome is *Mycgr3T108464*, and there are 6 paralogs. Comparing the DNA sequence of this gene against the 3D1 genome revealed an identical sequence, but the annotation of the gene in 3D7 and 3D1 differs; the 3D1 ortholog protein length is shorter, and the gene has 1 fewer exons. Our RNA sequence data suggest that the *ZT3D7_G3658* annotation may be incorrect; there were no reads mapping to the first, small exon of this gene. This suggests that the annotation of this gene may need manual curation before conducting further analyses. The gene was downregulated in YSB and MM in 3D1 compared to 3D7, demonstrating that transcription of this gene varies between the parents. Vacuoles play important roles in both osmotic stress responses (Richards *et al.* 2010) and virulence (Li *et al.* 2021). Under salt stress, the vacuoles can undergo fusion and fission, changing in size and number (Richards *et al.* 2010). *Vsp17* knockouts in *S. cerevisiae* had many small vacuoles but were resistant to osmotic stress and could sporulate (Köhrer and Emr 1993). We are unaware of any work that has investigated the role of *Vsp17* in the salt stress responses of filamentous fungi.

### Enrichment analysis of QTL unique to the KCl environment

We investigated if genes within QTL unique to the KCl environment were functionally enriched. We considered each cross separately and considered colony size-related traits and melanin-related traits separately (Tables 2 and 3: QTL no. ^S or M^). No KEGG pathway enrichment was detected. This may reflect the relatively low number of genes that were assigned a K number (3D7 34%; 1A5 49%). The GO enrichment analysis provided some interesting results. For QTL associated with colony size-related traits, 2 notable GO annotations were enriched. The first was the cell wall (GO: 0005618) that was enriched in QTLs in the 1A5 × 1E4 cross. Cell wall remodeling is an important response to osmotic stress, and this remodeling is mediated by the Hog1 and MAPK pathways (as described earlier). The cell wall GO is associated with 4 genes in the QTL intervals: ZT1A5_G201, ZT1A5_G1063, ZT1A5_G1074, and ZT1A5_G1774. A common annotation feature of the first 2 genes is they contain a glycoside hydrolase family 16, which is involved in degradation and remodeling of the cell wall. In rice blast (*Magnaporthe oryzae*), glycoside hydrolases II homolog (MoGls2) mutants showed defects in cell wall integrity, were less sensitive to salt stress, and had reduced virulence (Li *et al.* 2016). The other 2 genes with this GO term (ZT1A5_G1074 and ZT1A5_G1774) contain yeast PIR protein repeat. A number of yeast cell wall glycoproteins are characterized by the presence of internal repeats (PIR, tandem repeats of 18–19 aa residues), and these are important in yeast response to multiple environmental stressors and antifungal agents (Mazáň *et al.* 2008). The second notable GO enrichment was for GO terms related to DNA repair (GO: 0006281: nucleotide-excision repair, double-strand break repair via nonhomologous end joining). DNA repair genes can be upregulated in the presence of a variety of environmental stressors and are widely considered an important response to stress (Hohmann 2002). Six genes have these GO annotations (ZT3D7_G5293, ZT3D7_G3673, ZT3D7_G3591, ZT3D7_G5301, ZT3D7_G5305, and ZT3D7_G5361). Three of these genes (ZT3D7_G5293, ZT3D7_G3673, and ZT3D7_G53601) have multiple GO annotations, of which 3 were also enriched: DNA binding (GO: 0043564), damaged DNA binding (GO: 0003684), and telomeric DNA binding (GO: 0042162). Another 3 genes with enriched GO annotations were in or adjacent to the large LOD QTL on chromosome 3 (Fig. 4; Table 4) in the 3D7 × 3D1 cross (ZT3D7_G3648, ZT3D7_G3652, and ZT3D7_G3659, Supplementary Table 7). Two have predicted GTPase activity (GO: 0003924; ZT3D7_G3648 and ZT3D7_G3659). GTPases are involved in many functions in fungi, including during plant-fungal interactions. A well-studied GTPase family, *Ras*, has been shown to be involved in response to osmotic stress in *Beauveria bassiana* and *Botrytis cinerea* (Dautt-Castro *et al.* 2021). Fungal treatments targeting Ras proteins are being tested to control human pathogens with promising results (LeBlanc *et al.* 2020). The other gene in the interval, ZT3D7_G3652, has predicted superoxide dismutase activity (GO: 0004784). Genes in this family are known to play an important role in defending cells against reactive oxygen species, and in the stripe rust fungus (*P. striiformis* f. sp. *tritici*), this can help guard against plant defenses and facilitate infection (Zheng *et al.* 2020).

There was also a notable GO term enriched for melanin-related QTL: glutathione biosynthetic process (GO: 0006750). This GO term was associated with 3 genes within QTLs: ZT3D7_G1409, ZT3D7_G1830, and ZT3D7_G8325. Glutathione is a metabolite that plays a major role in response to stress, morphogenesis, and virulence in fungi (Wangsanut and Pongpom 2022). In *C. neoformans*, a glutathione gene mutant (gsh2Δ) had increased sensitivity to salt (Berndt and Lillig 2017), and *Schizosaccharomyces pombe* grown in vitro under multiple stressors, including osmotic stress, had increased expression of the glutathione reductase gene (*pgr1*) (Lee *et al.* 1997). Our results suggest that glutathione may also be important in osmotic stress response in *Z. tritici*. All the genes highlighted in our GO enrichment analysis may be useful targets for further investigation to understand the molecular response of *Z. tritici* to salt stress but may also provide interesting targets for fungicide development.

### QTL reproducibility

This data set allowed us to explore the repeatability of previously identified QTL from other published data sets. One earlier data set used the same mapping populations but different experimental conditions and treatments, with a different reference genome and different genetic markers and map information (Lendenmann *et al.* 2014, 2015). A second earlier data set used the same isolates and experimental conditions but different treatments and different marker and map information (Zhong *et al.* 2021). Comparisons across these 3 data sets yielded several consistent QTL. Our analyses confirmed that a region on chromosome 11 in the 3D7 × 3D1 cross (Table 2, QTL no. 11) is associated with variation in melanization and growth in multiple environments (e.g. under fungicide stress in Lendenmann *et al.* 2015; under oxidative stress in Zhong *et al.* 2021). This QTL contains the *Zmr1* TF that was shown to regulate melanin production (Krishnan *et al.* 2018). We hypothesize that *Zmr1* also explains the variation we observed under salt stress. A QTL associated with multiple growth and melanin traits in the 1A5 × 1E4 cross on chromosome 8 (Table 3, QTL no. 13) overlaps with a previously identified QTL on chromosome 8 that was associated with colony growth and tolerance to oxidative stress (Zhong *et al.* 2021). These findings illustrate that many of our QTL are reproducible across experiments and increases our confidence that we have identified biologically meaningful genomic regions using our automated image analyses.

## Supplementary Material

jkad226_Supplementary_Data

## Data Availability

Data are available at https://www.research-collection.ethz.ch/handle/20.500.11850/550424. All metadata, code, and details of the analysis are available at https://github.com/jessstapley/QTL-mapping-Z.-tritici. Supplemental material available at G3 online.
