# Supplementary material for: Quantitative trait locus mapping of osmotic stress response in the fungal wheat pathogen Zymoseptoria tritici
Source: G3 (Bethesda). 2023 Sep 29;13(12):jkad226. doi: 10.1093/g3journal/jkad226 (PMC10700024; doi:10.1093/g3journal/jkad226)
Supplement: jkad226_Supplementary_Data [file jkad226_supplementary_data.zip › Supplemental_Material_Legends_G3-2023-404429.docx]

Supplementary Legend

**Supplementary Figures**

Figure S1. Correlation of growth rate and melanisation rate in control and KCl environment.

Figure S2. Correlation of mean radius and mean grey value at 12 dpi in control and KCl environment.

Figure S3. LOD plots from interval mapping for all traits in the 3D7x3D1 cross.

Figure S4. LOD plots from interval mapping for all traits in the 1A5x1E4 cross.

**Supplementary Tables**

Table S1. Linkage map summary.

Table S2. Summary of phenotypic data.

Table S3. Trait correlations in 3D7x3D1 cross.

Table S4. Trait correlations in 1A5x1E4 cross.

Table S5. Results of the GO enrichment analysis for 3D7x3D1 cross.

Table S6. Results of the GO enrichment analysis for 1A5x1E4 cross.

Table S7. GO enrichment genes.

Table S8. QTL interval on Chr 3 (QTL #3) in 3D7x3D1 cross in detail.

Table S9. Results of the SNP effect analysis for the QTL interval on Chr 3 (QTL #3).
