# Supplementary figures and images for: Quantitative trait locus mapping of osmotic stress response in the fungal wheat pathogen Zymoseptoria tritici
Source: G3 (Bethesda). 2023 Sep 29;13(12):jkad226. doi: 10.1093/g3journal/jkad226 (PMC10700024; doi:10.1093/g3journal/jkad226)

1A5x1E4

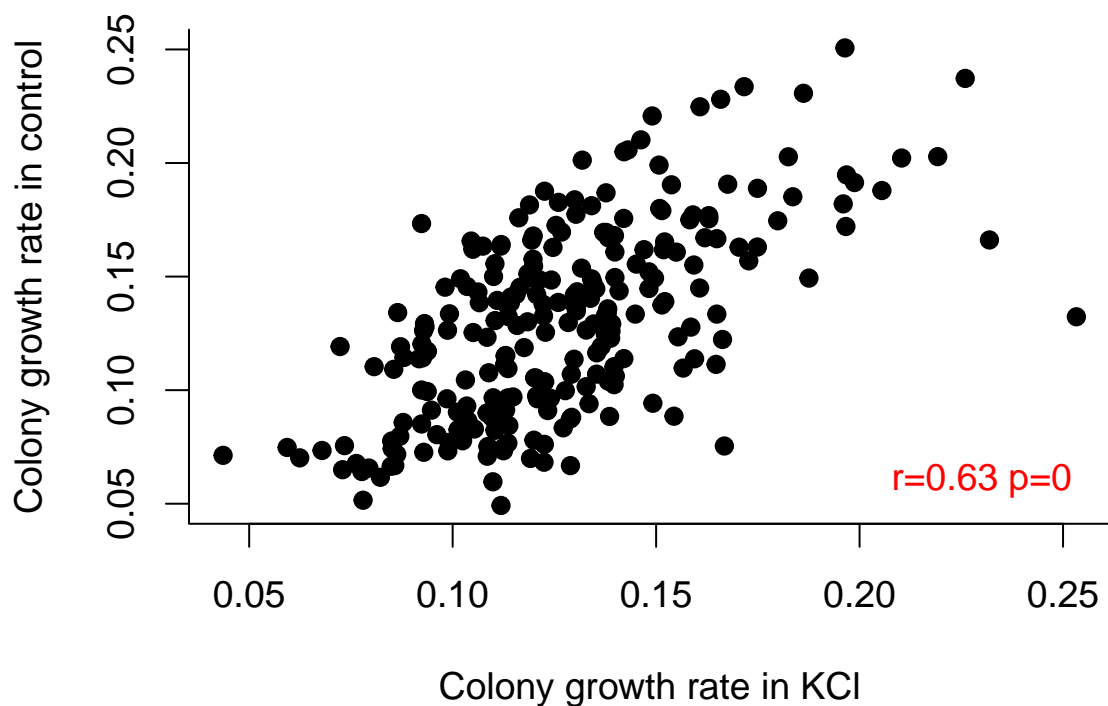

1A5x1E4

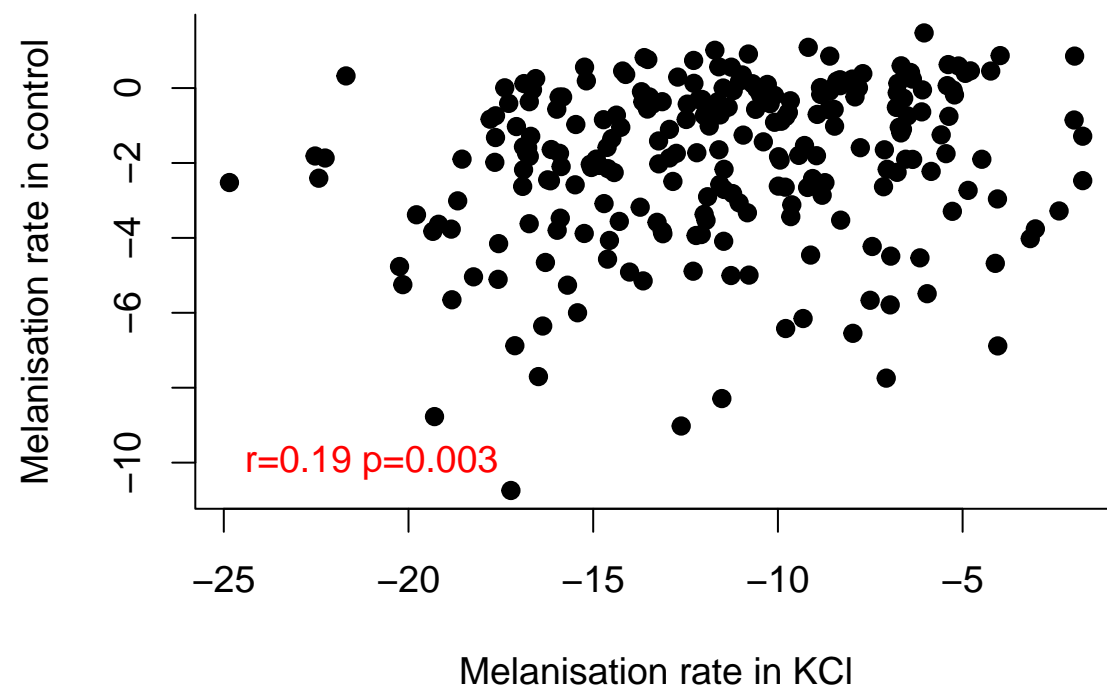

3D7x3D1

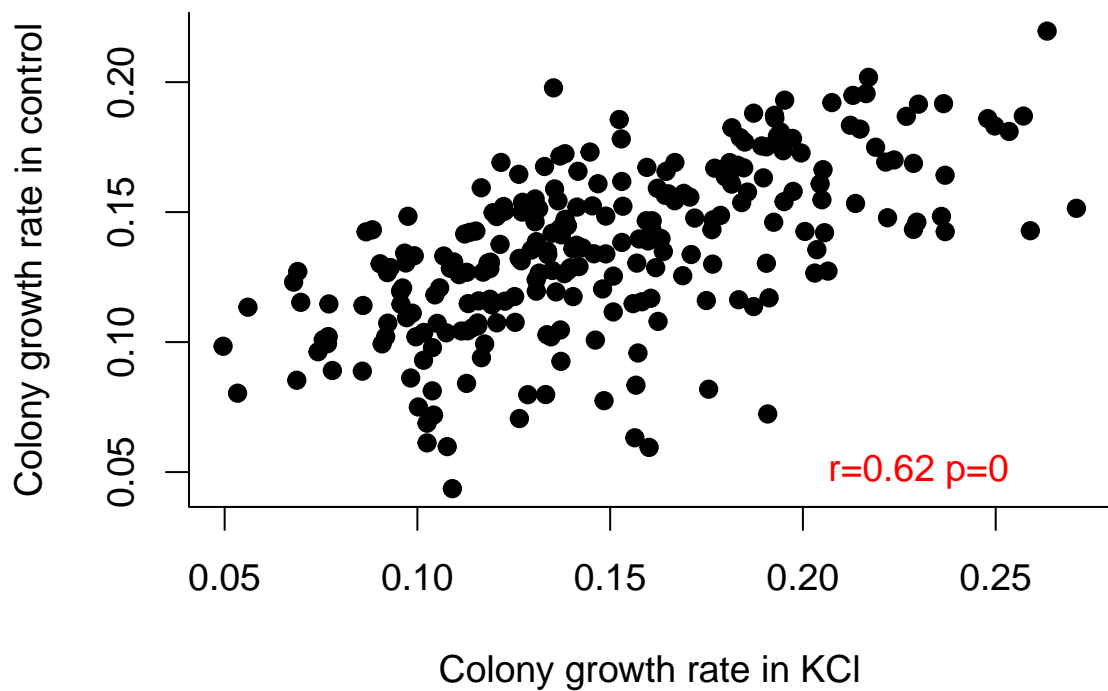

3D7x3D1

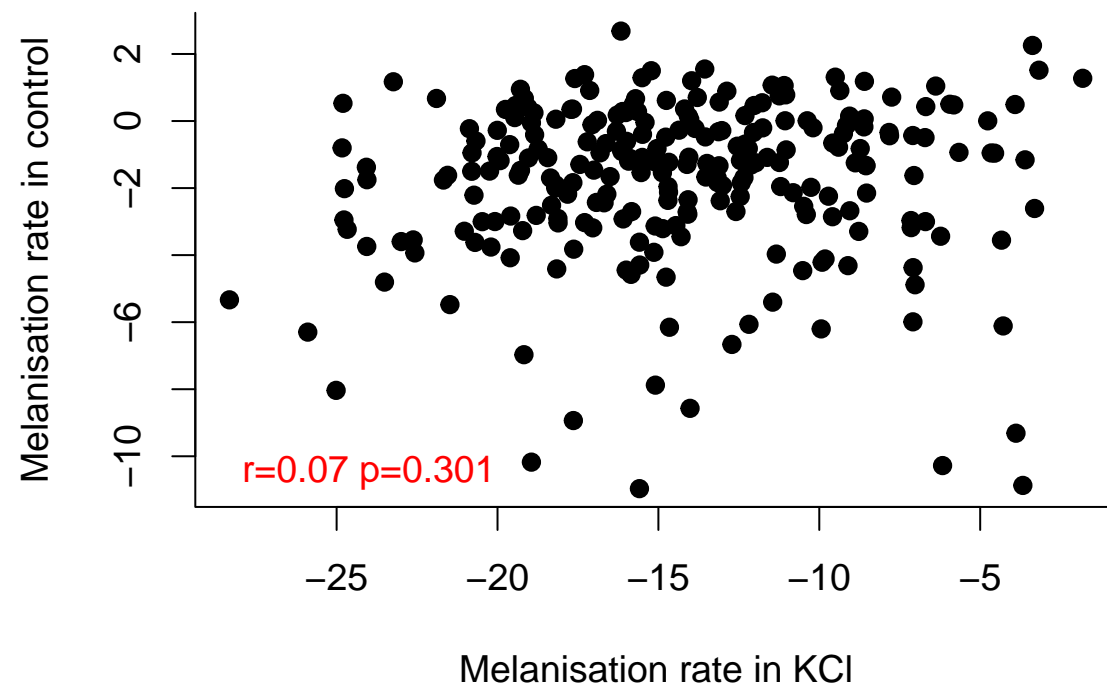

Supplement: jkad226_Supplementary_Data [file jkad226_supplementary_data.zip › Figure_S1_G3-2023-404429.pdf]

1A5x1E4

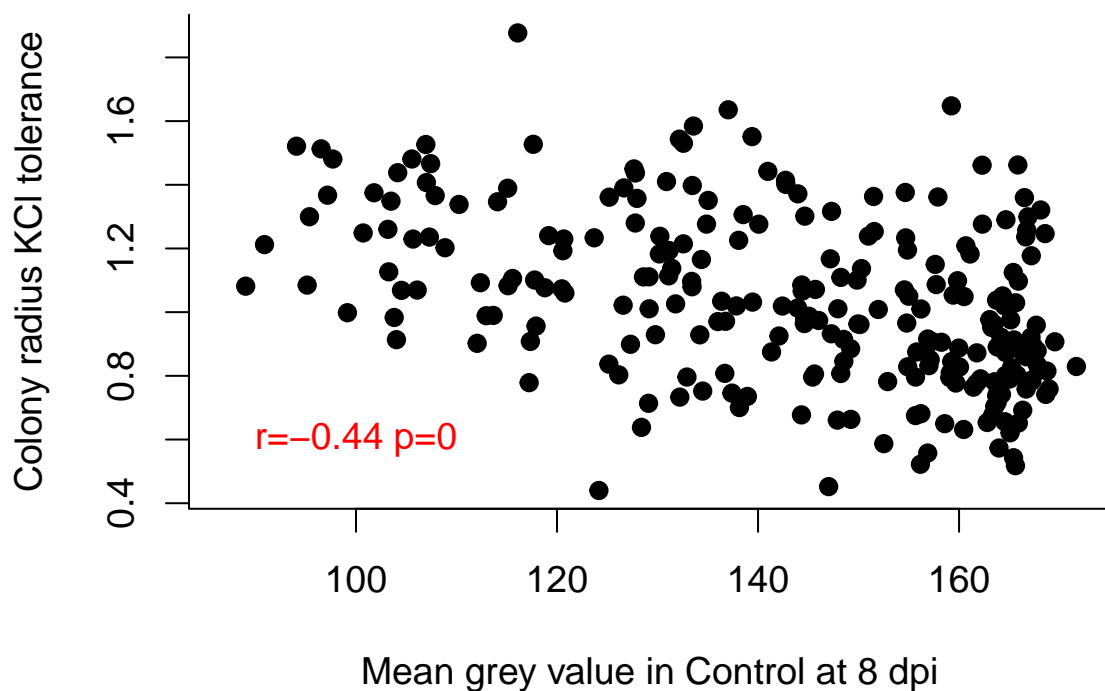

1A5x1E4

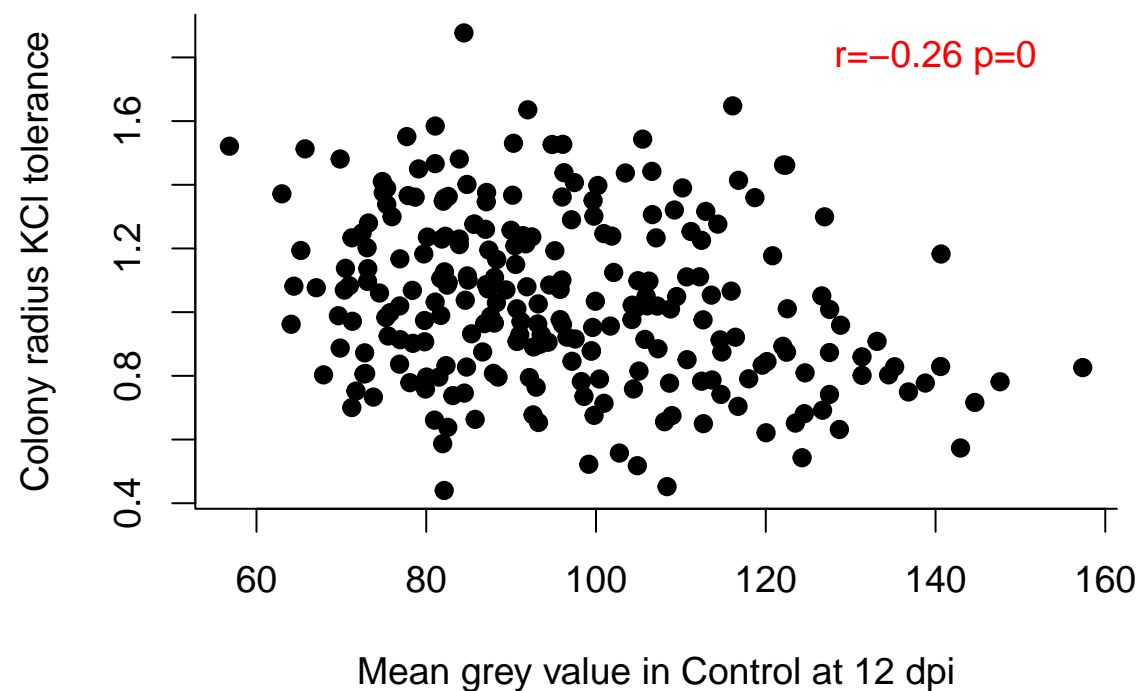

3D1x3D7

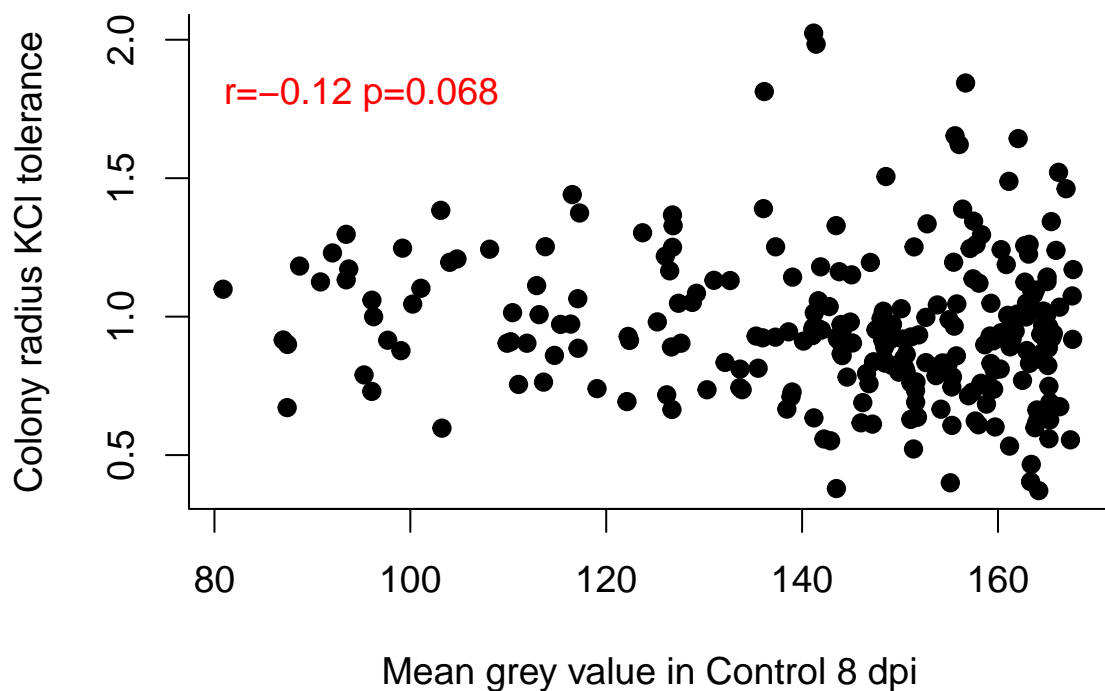

3D1x3D7

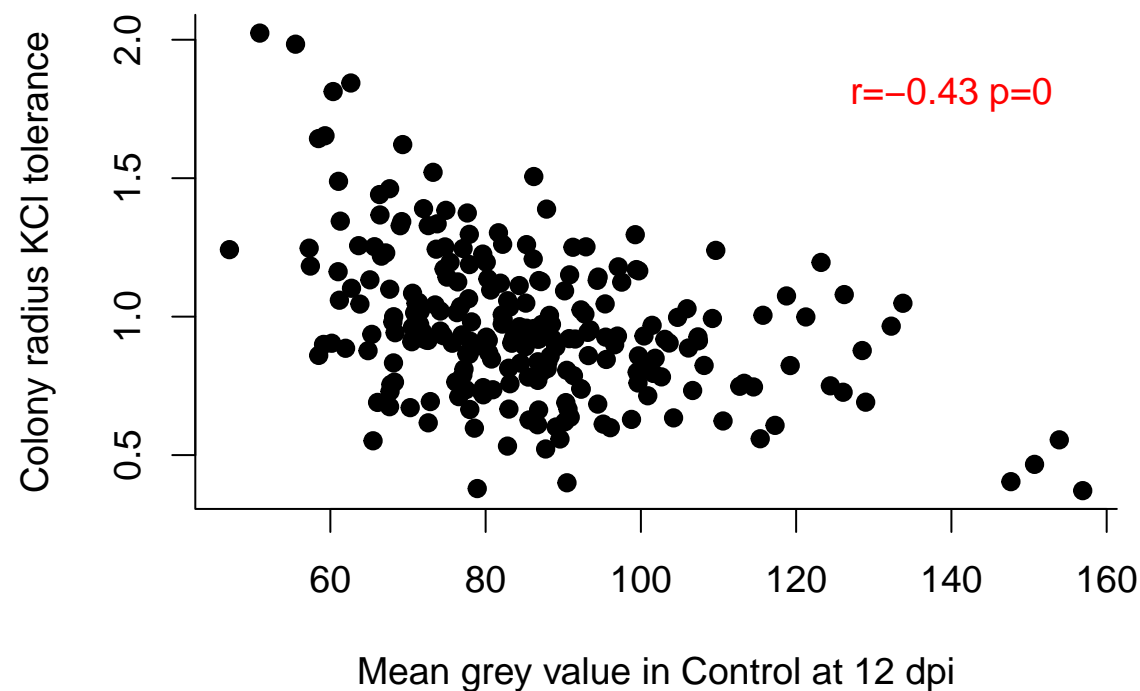

Supplement: jkad226_Supplementary_Data [file jkad226_supplementary_data.zip › Figure_S2_G3-2023-404429.pdf]

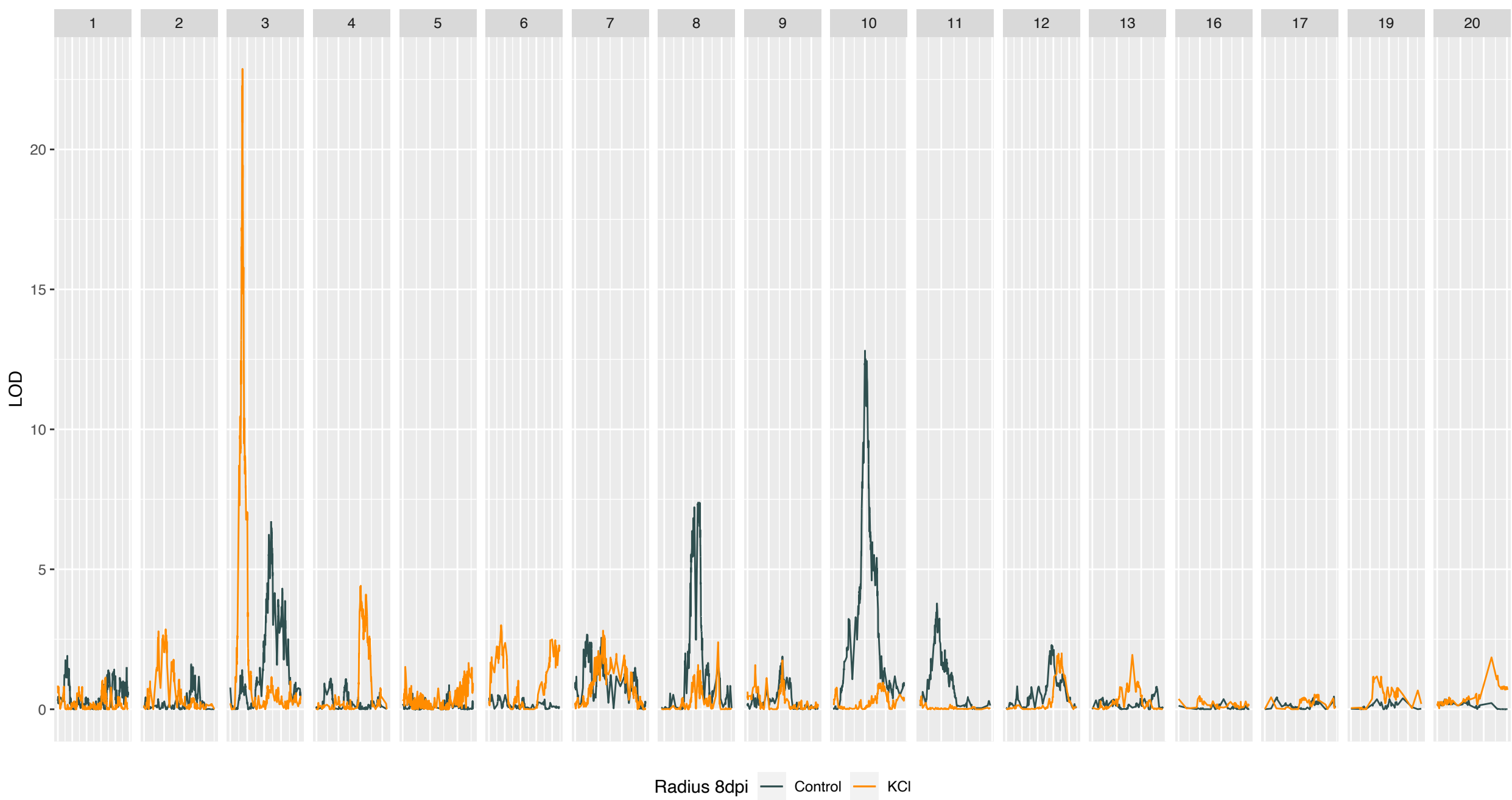

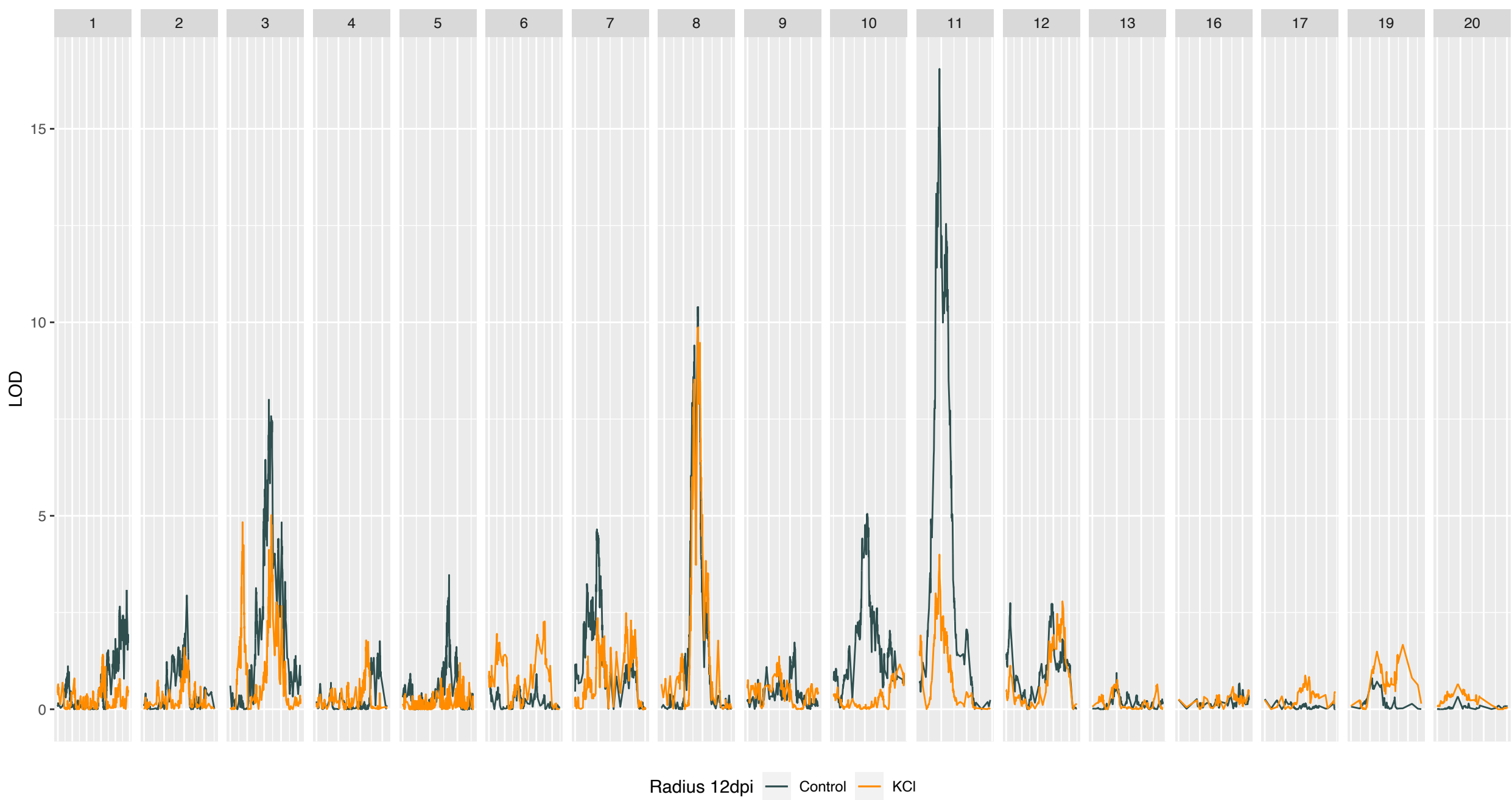

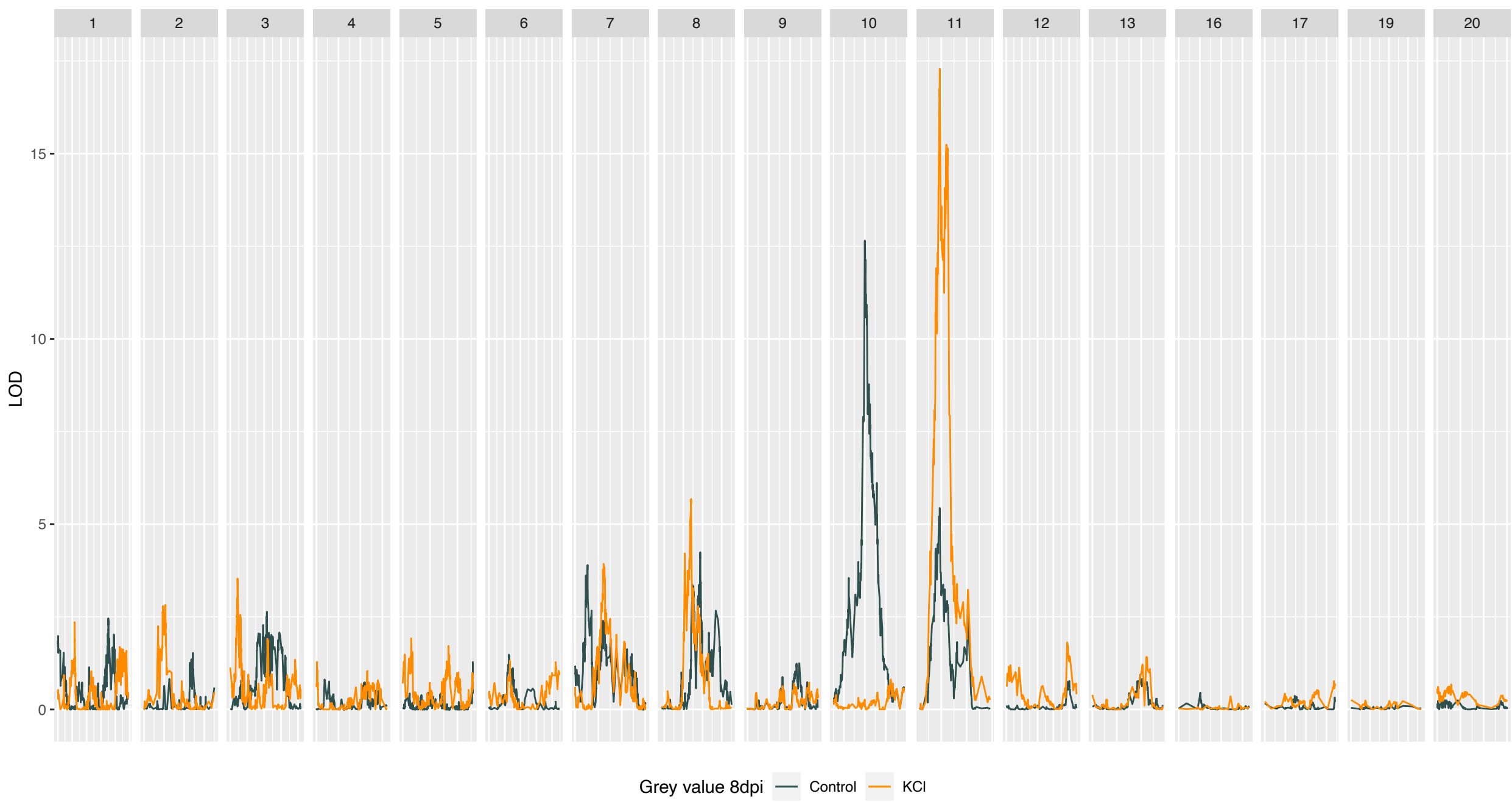

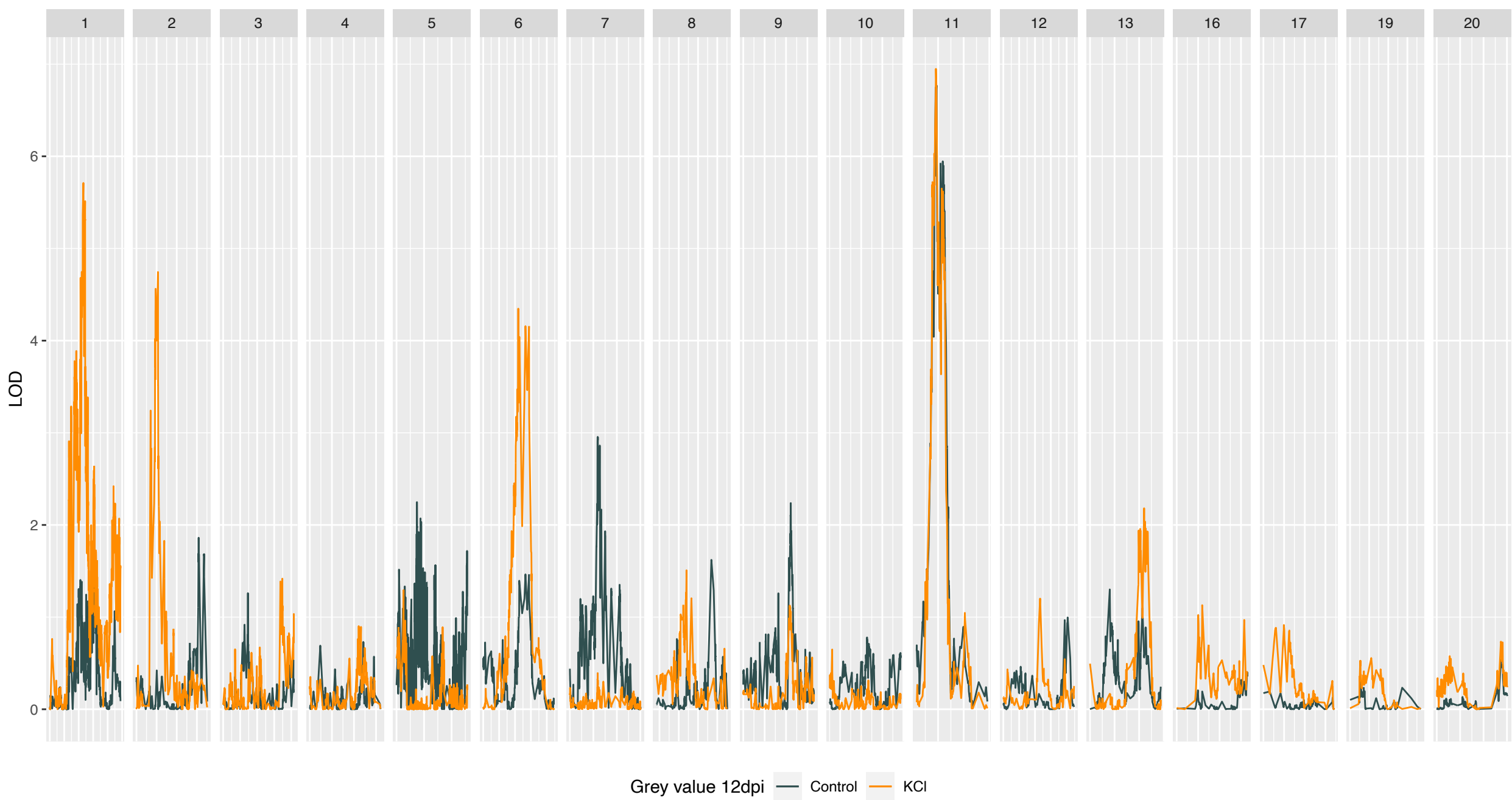

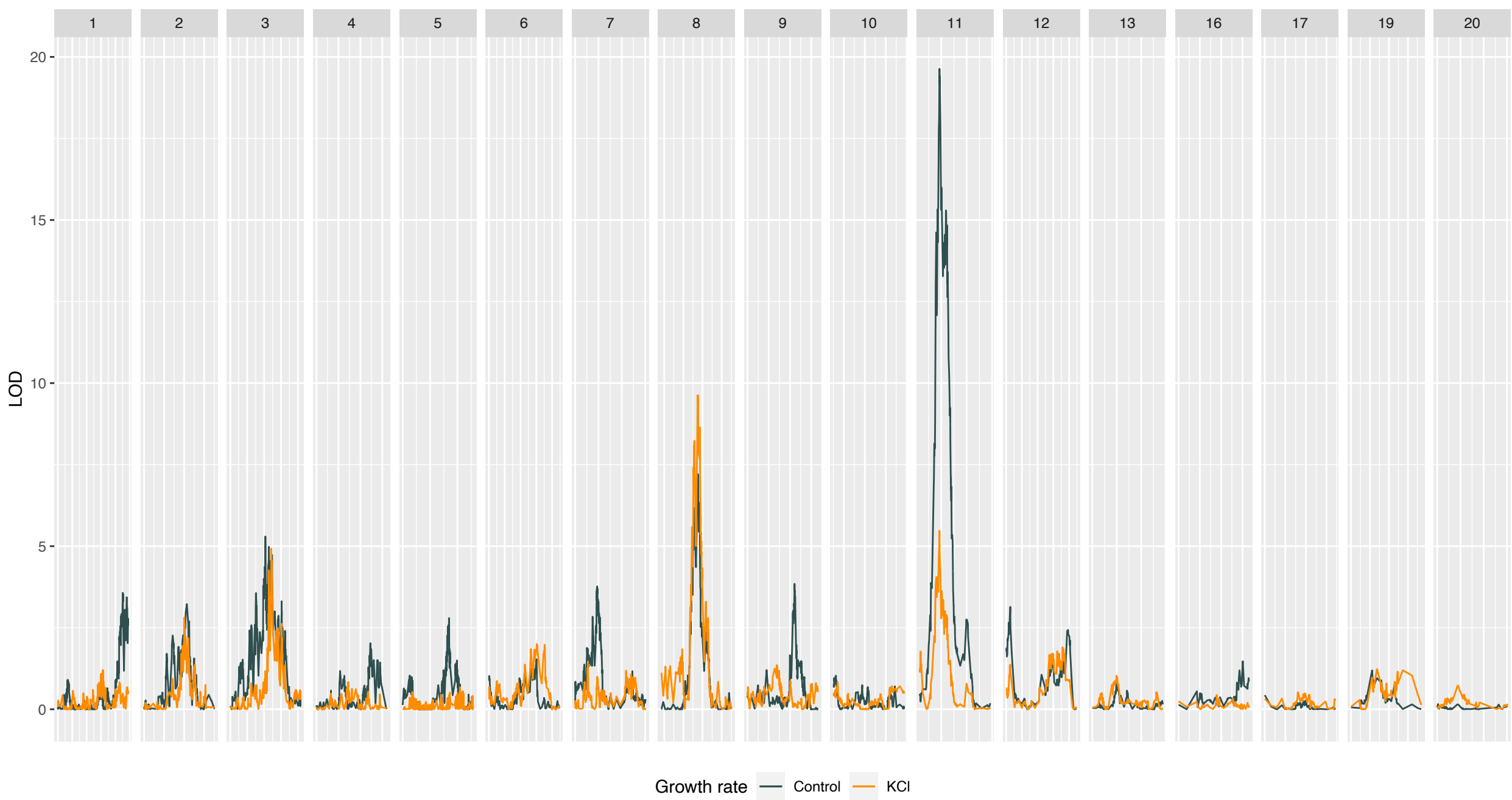

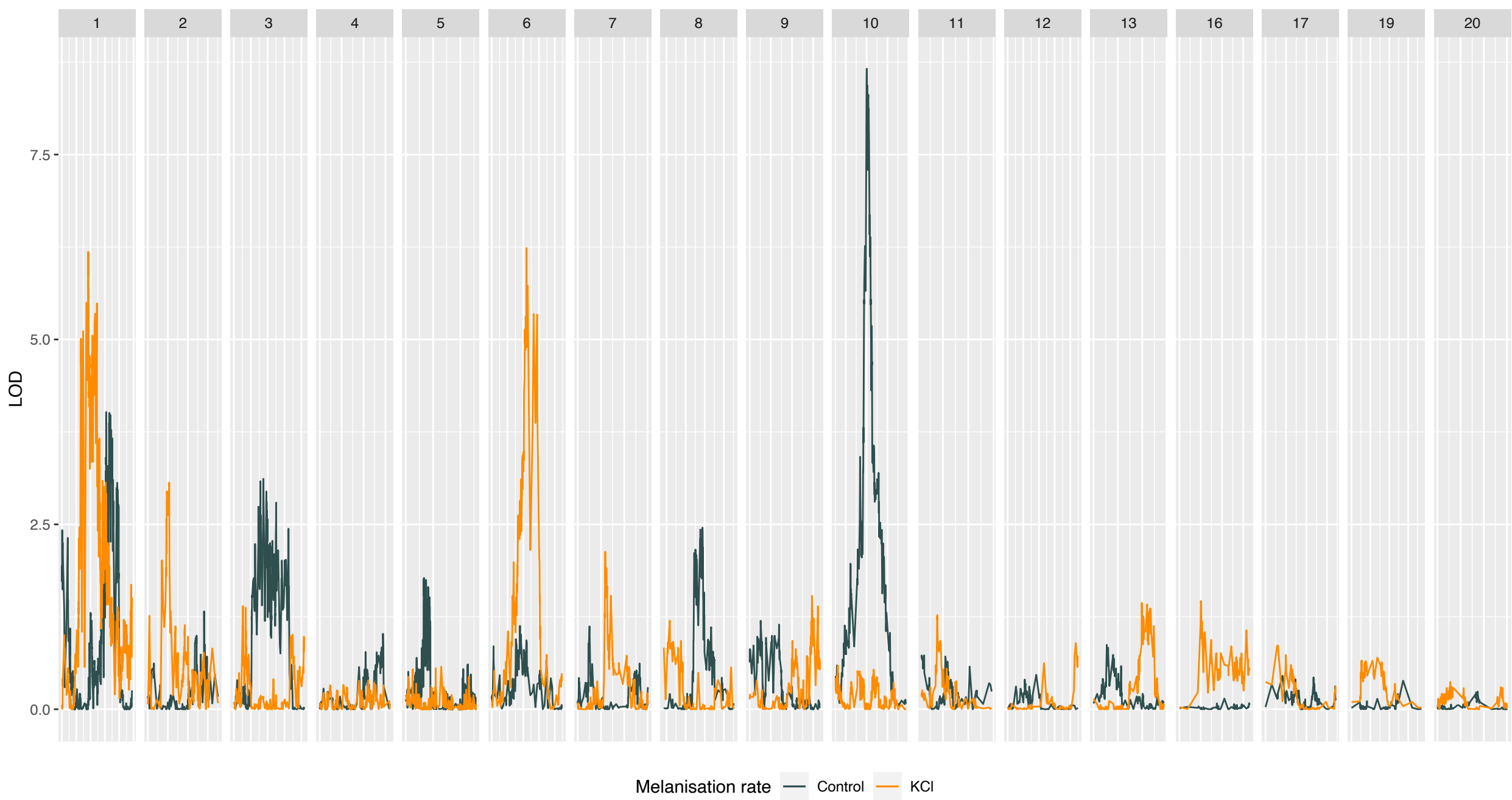

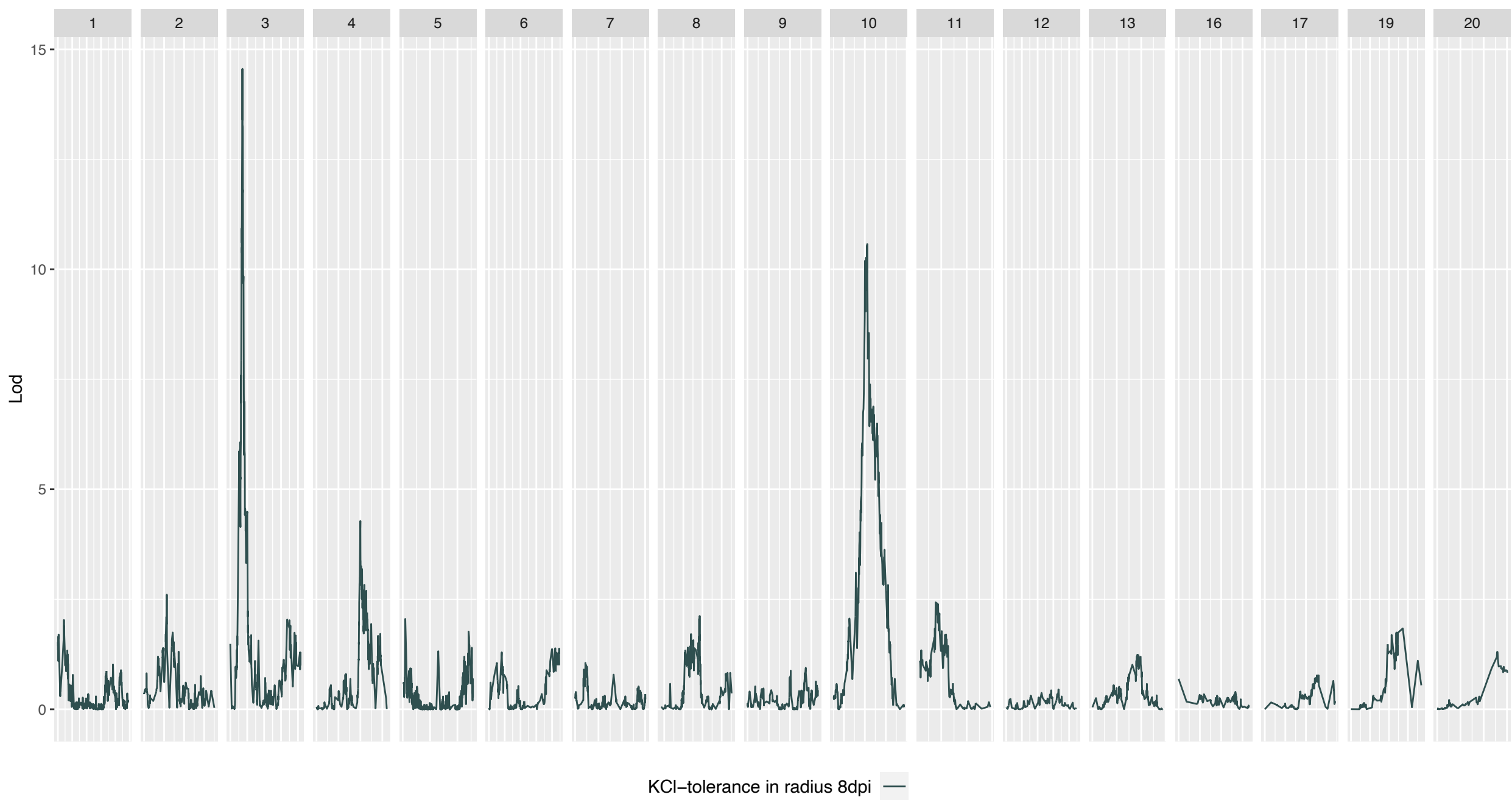

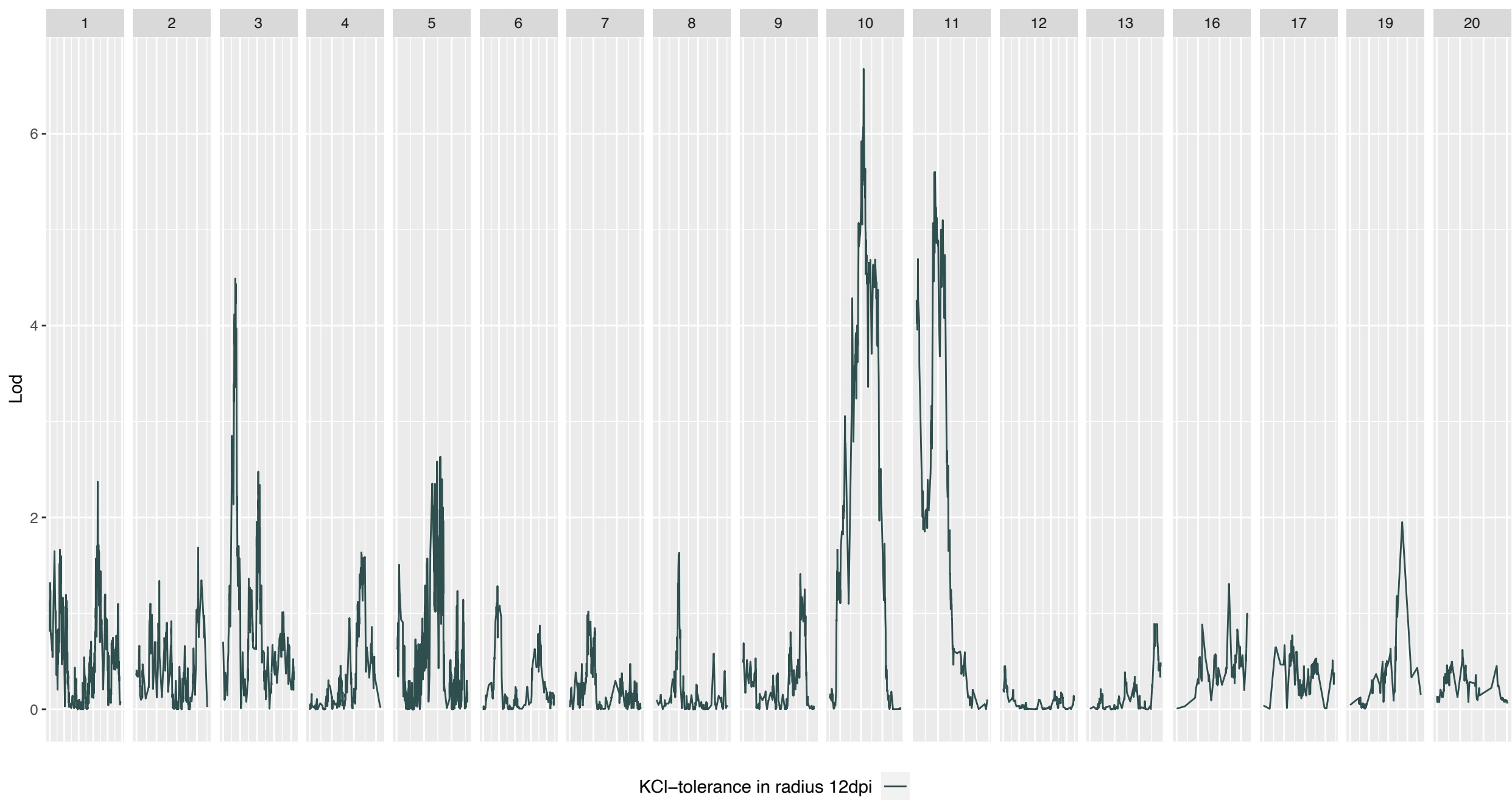

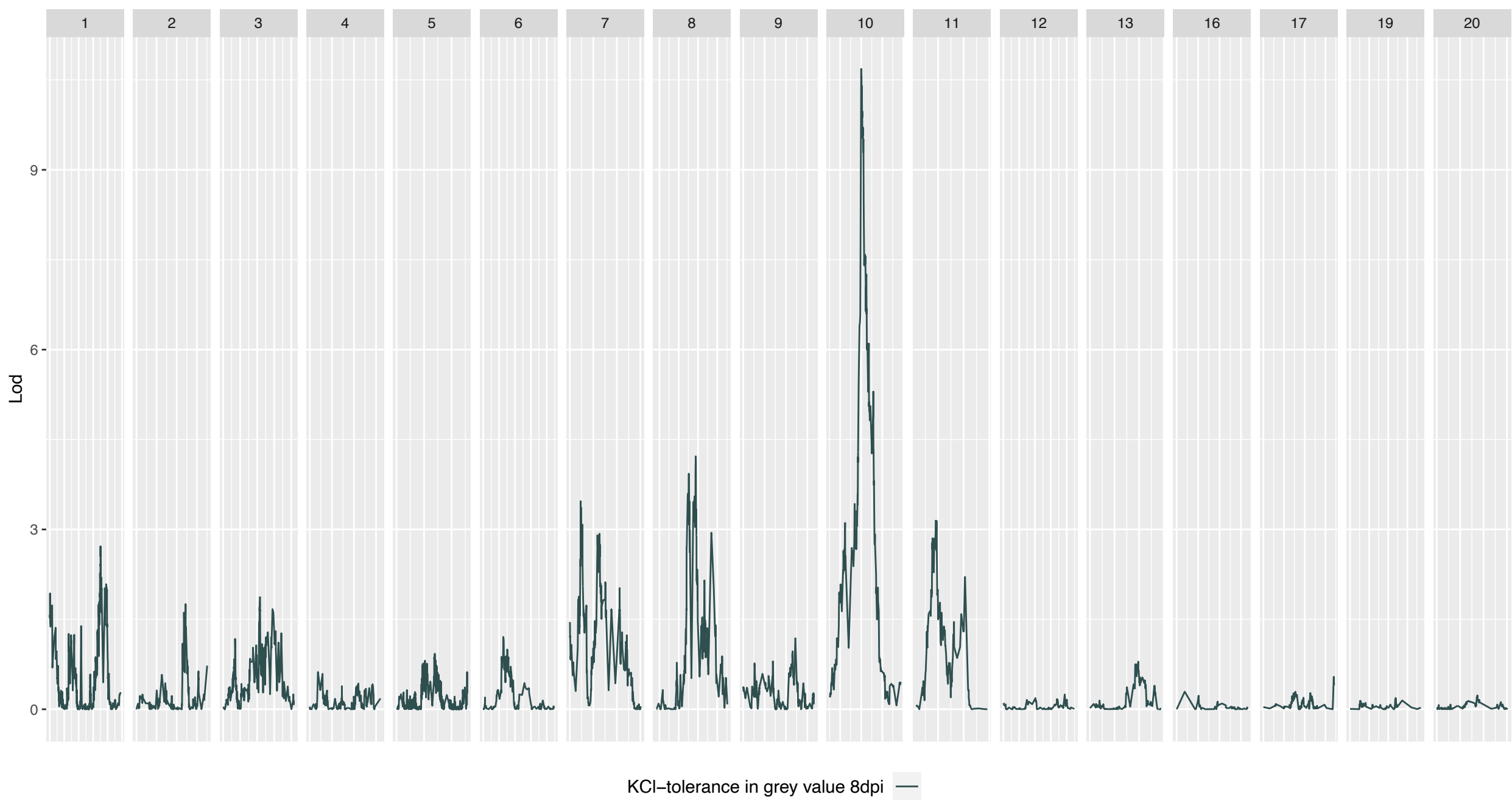

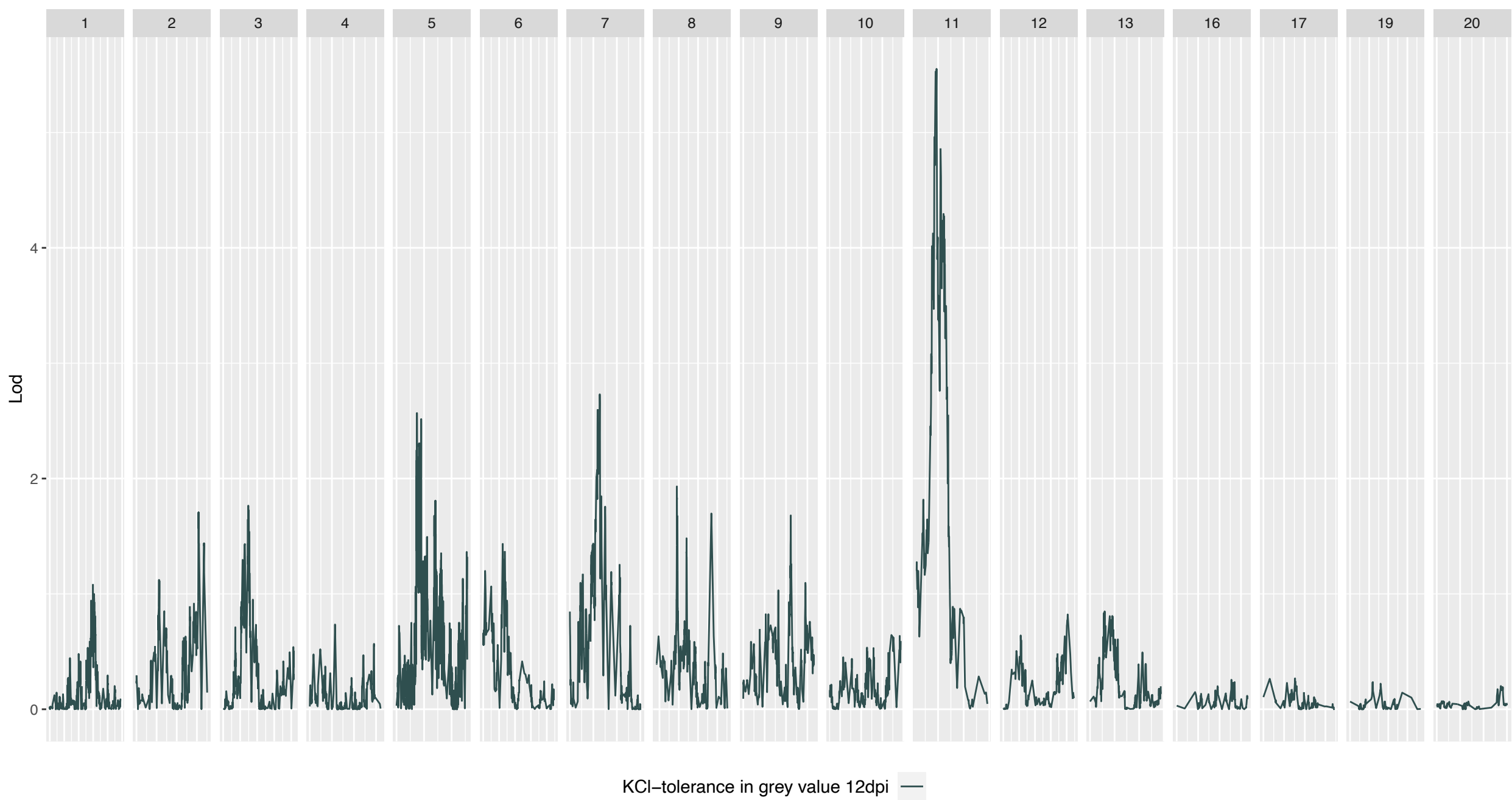

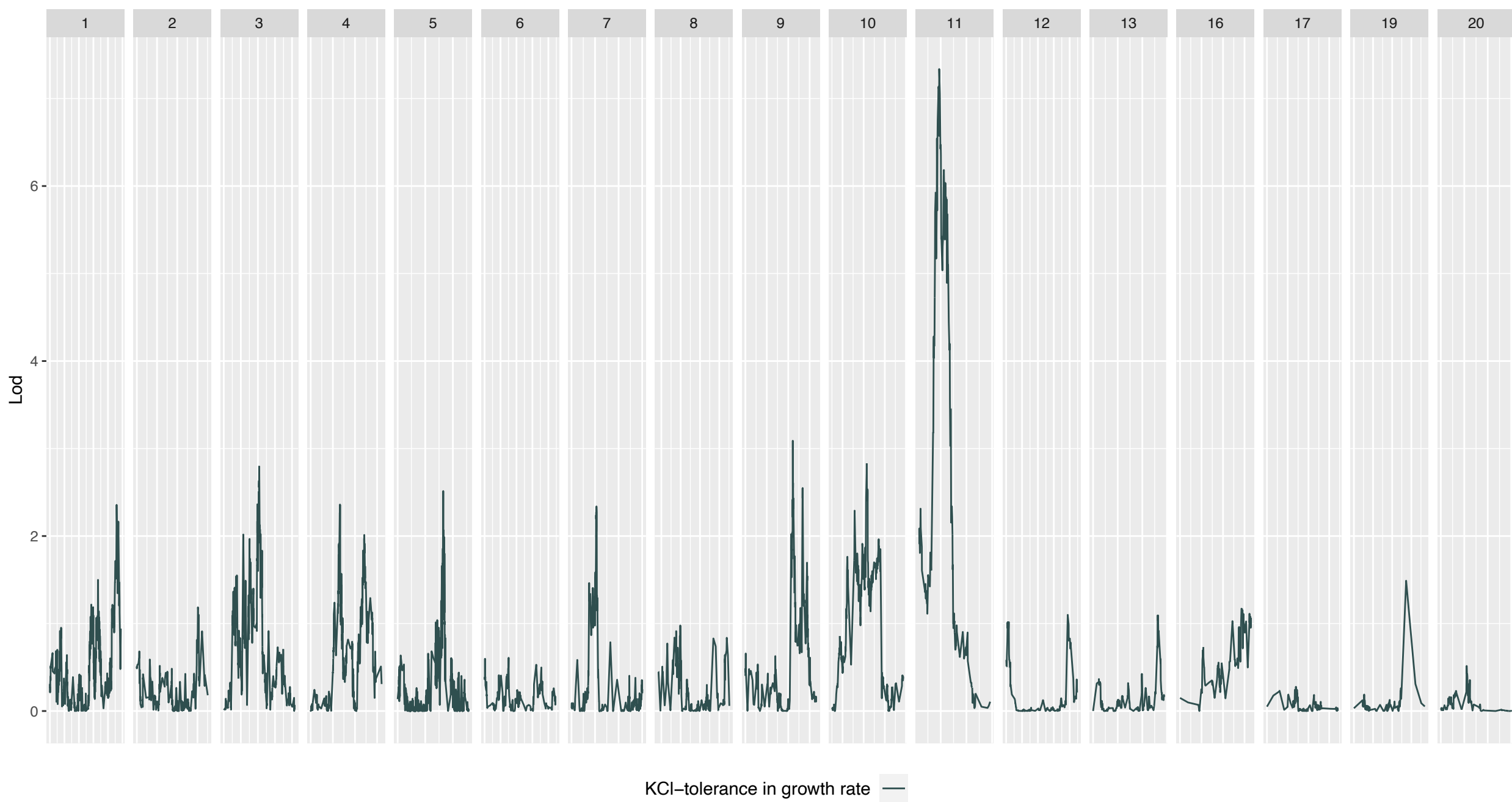

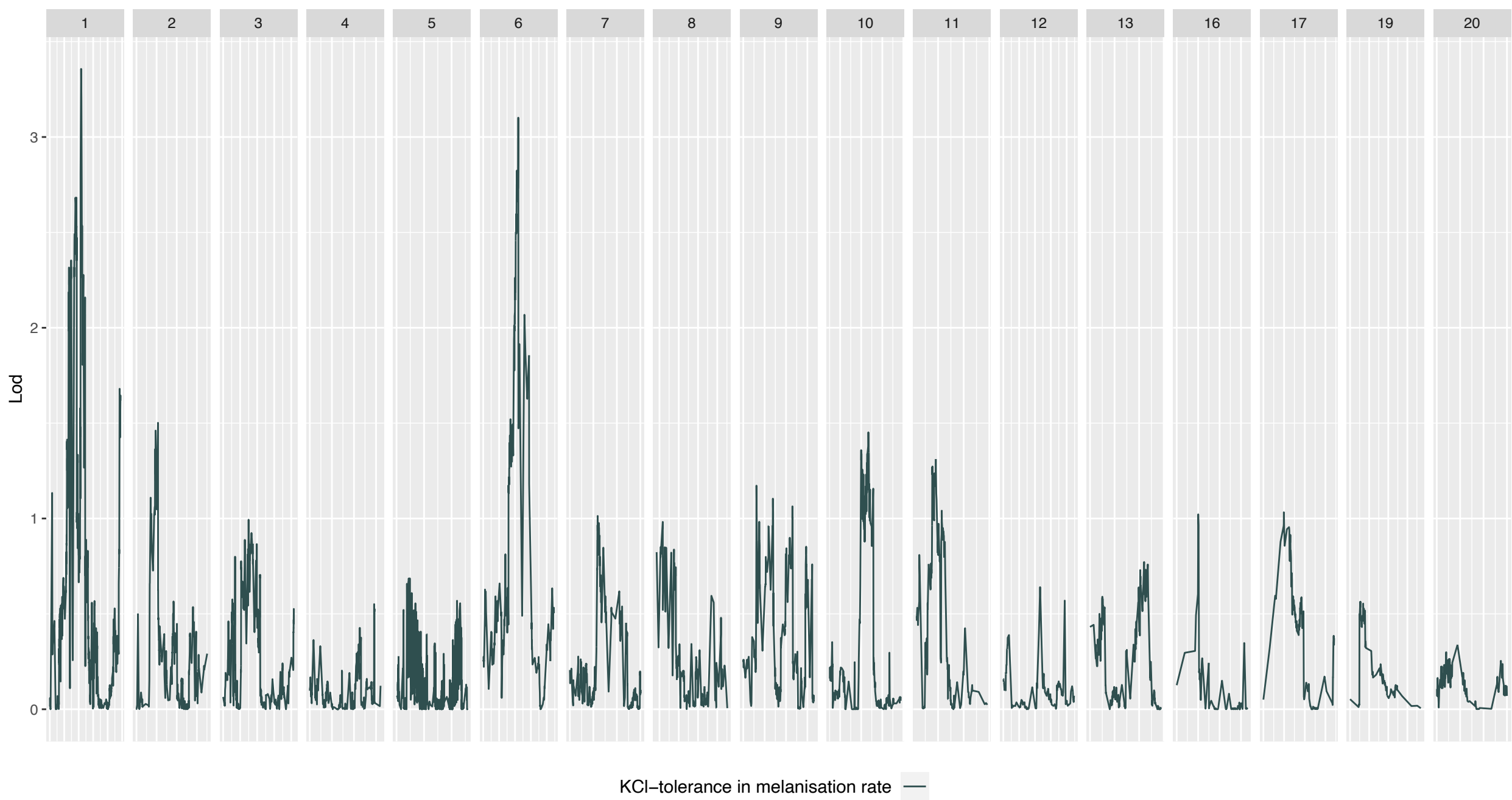

Supplement: jkad226_Supplementary_Data [file jkad226_supplementary_data.zip › Figure_S3_G3-2023-404429.pdf]

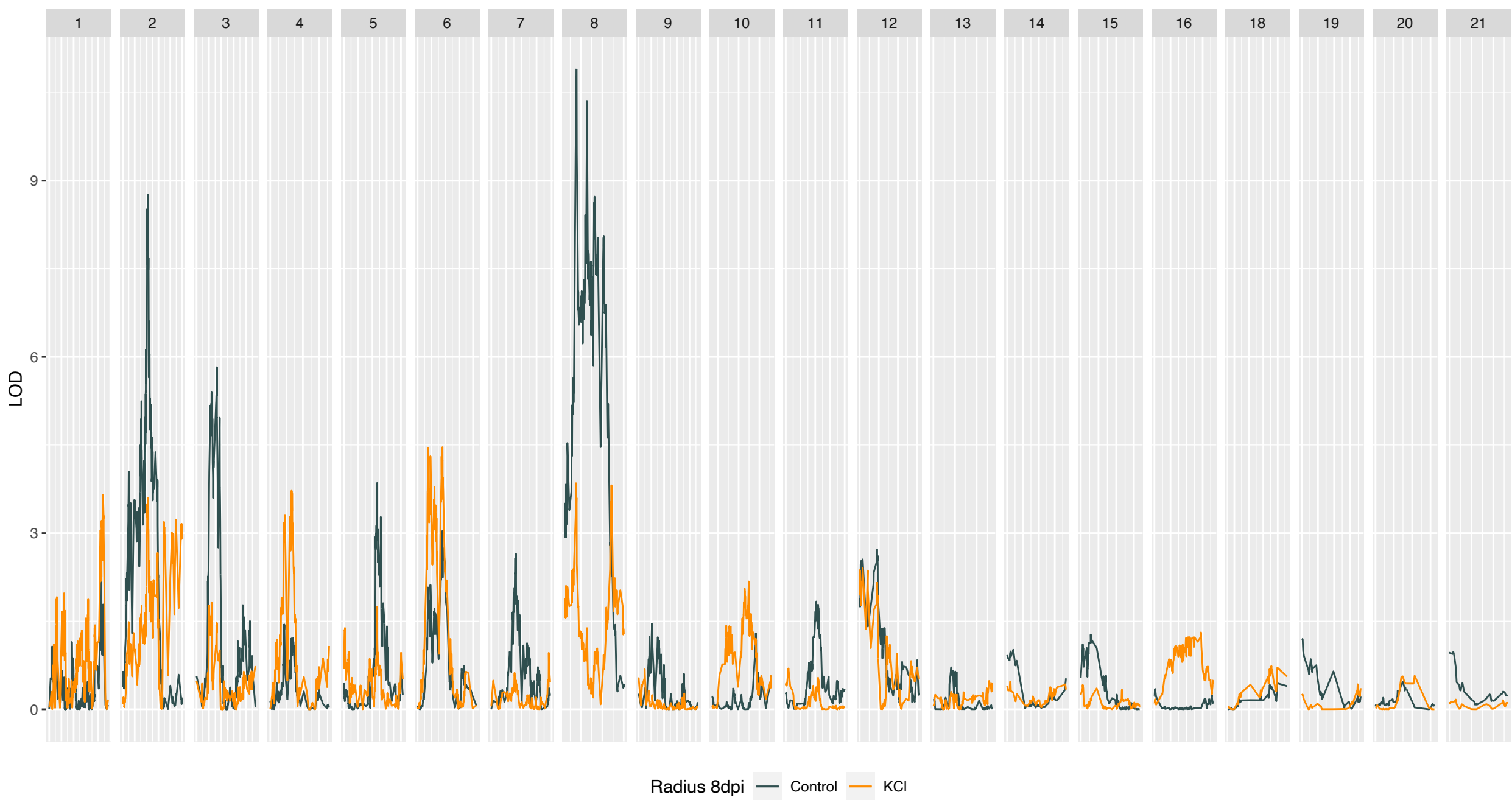

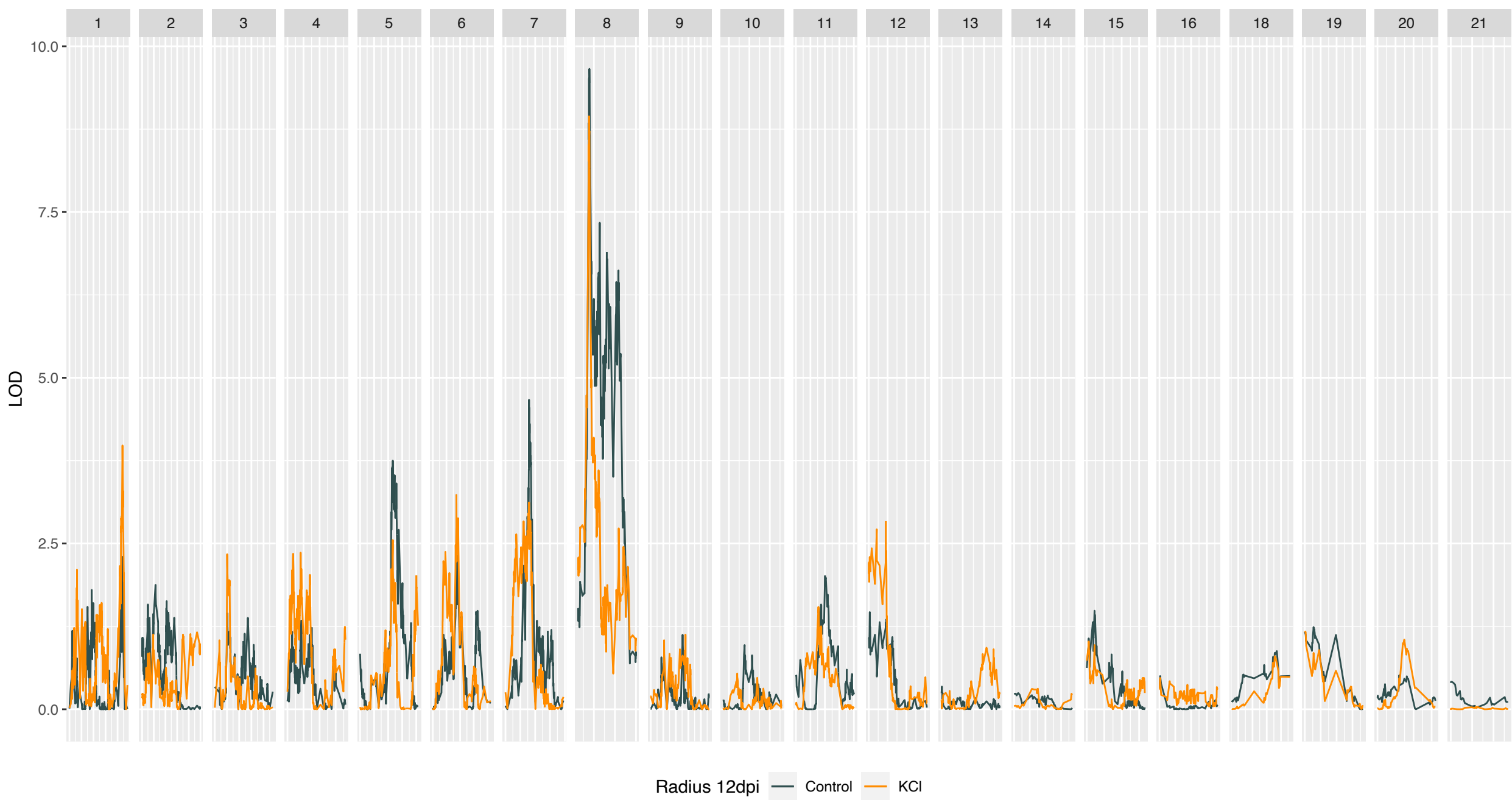

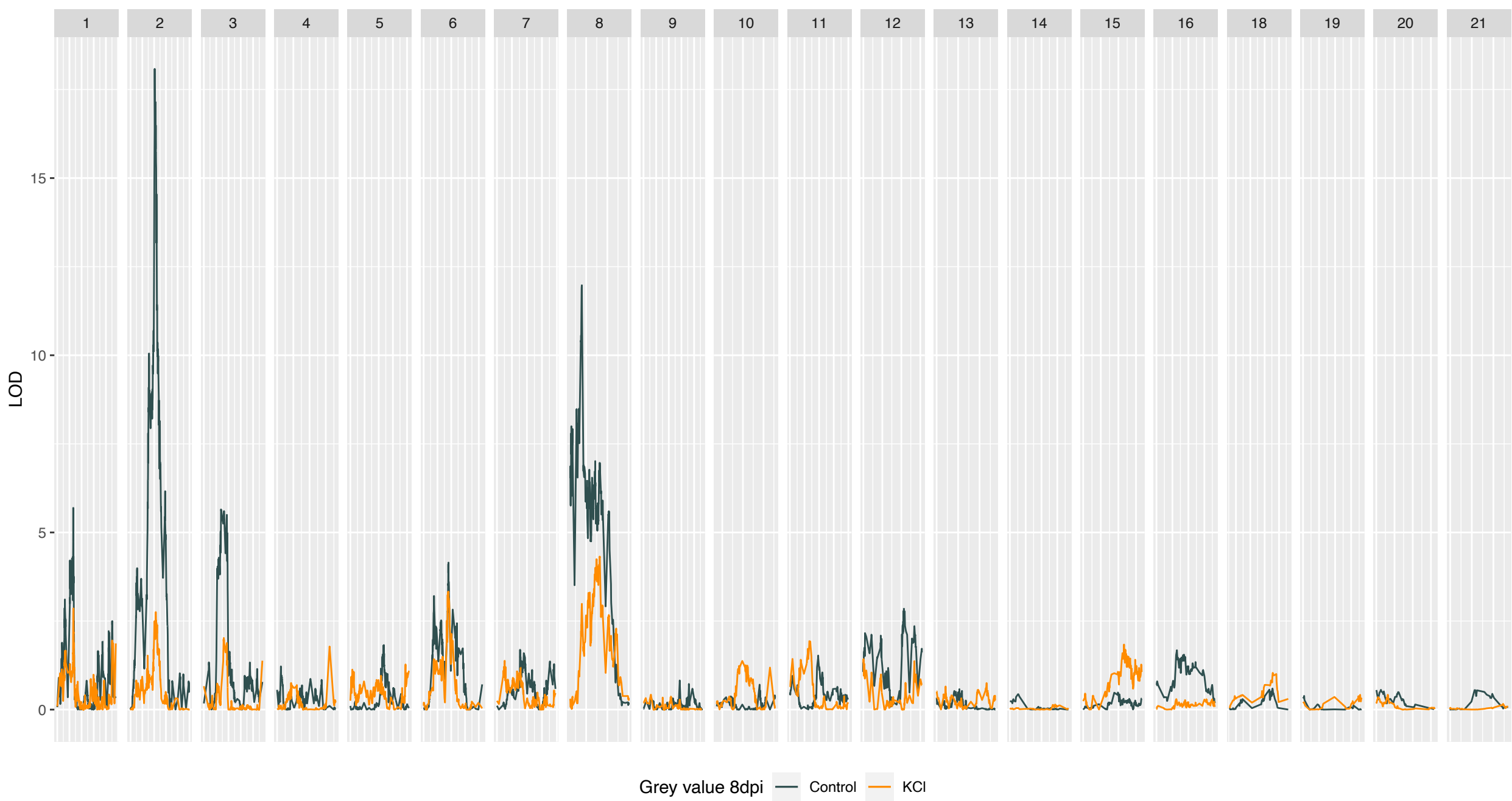

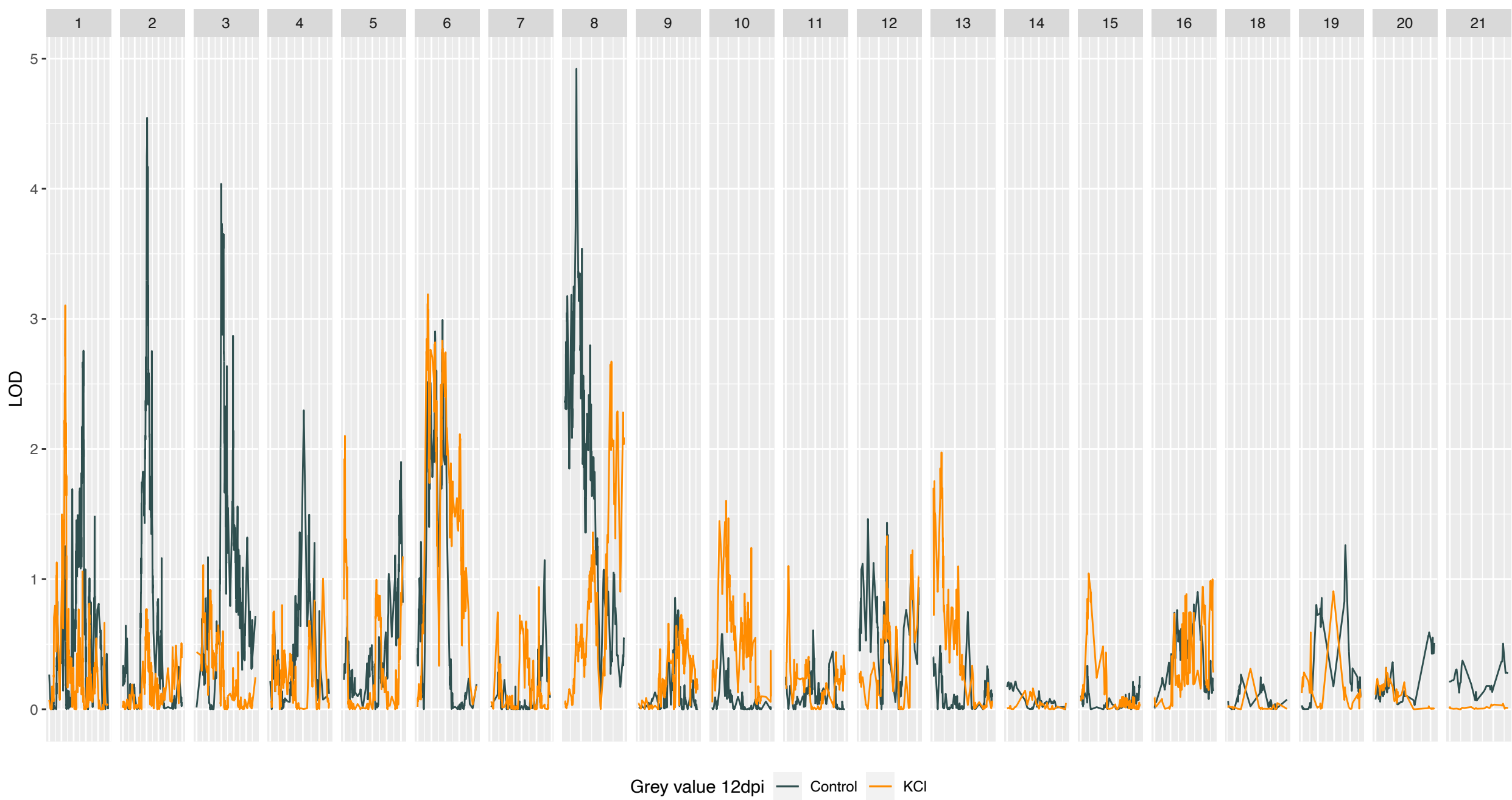

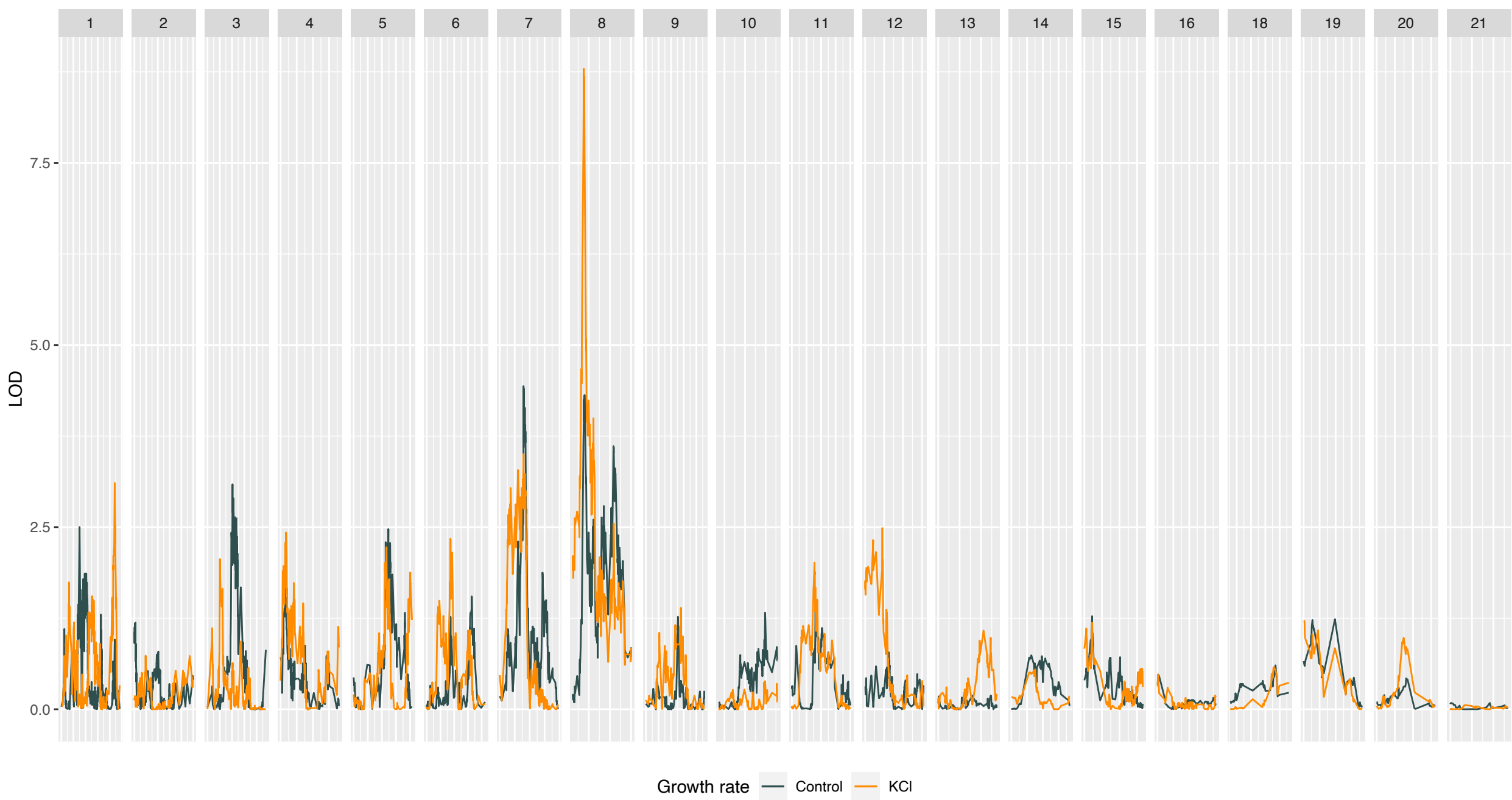

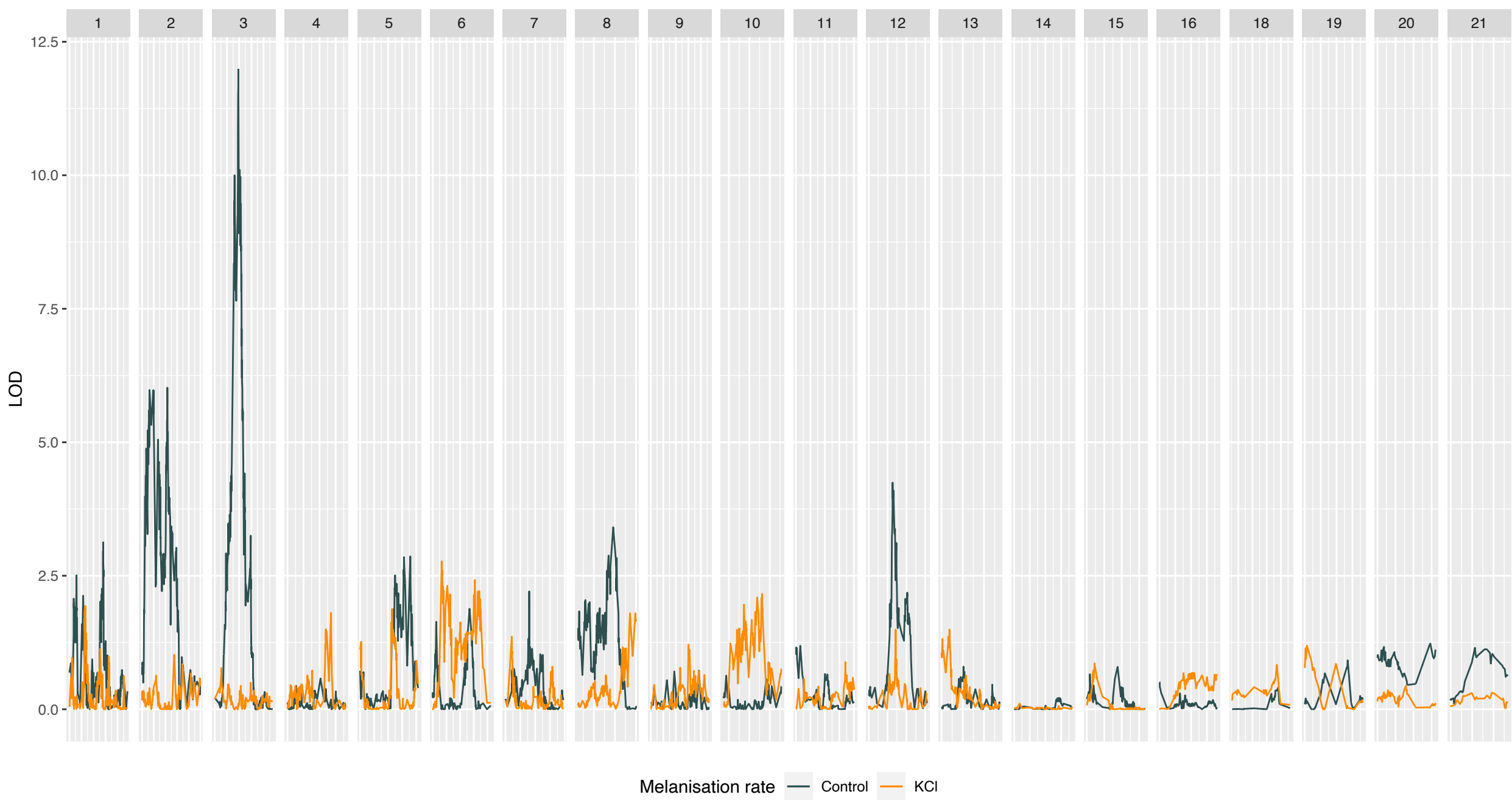

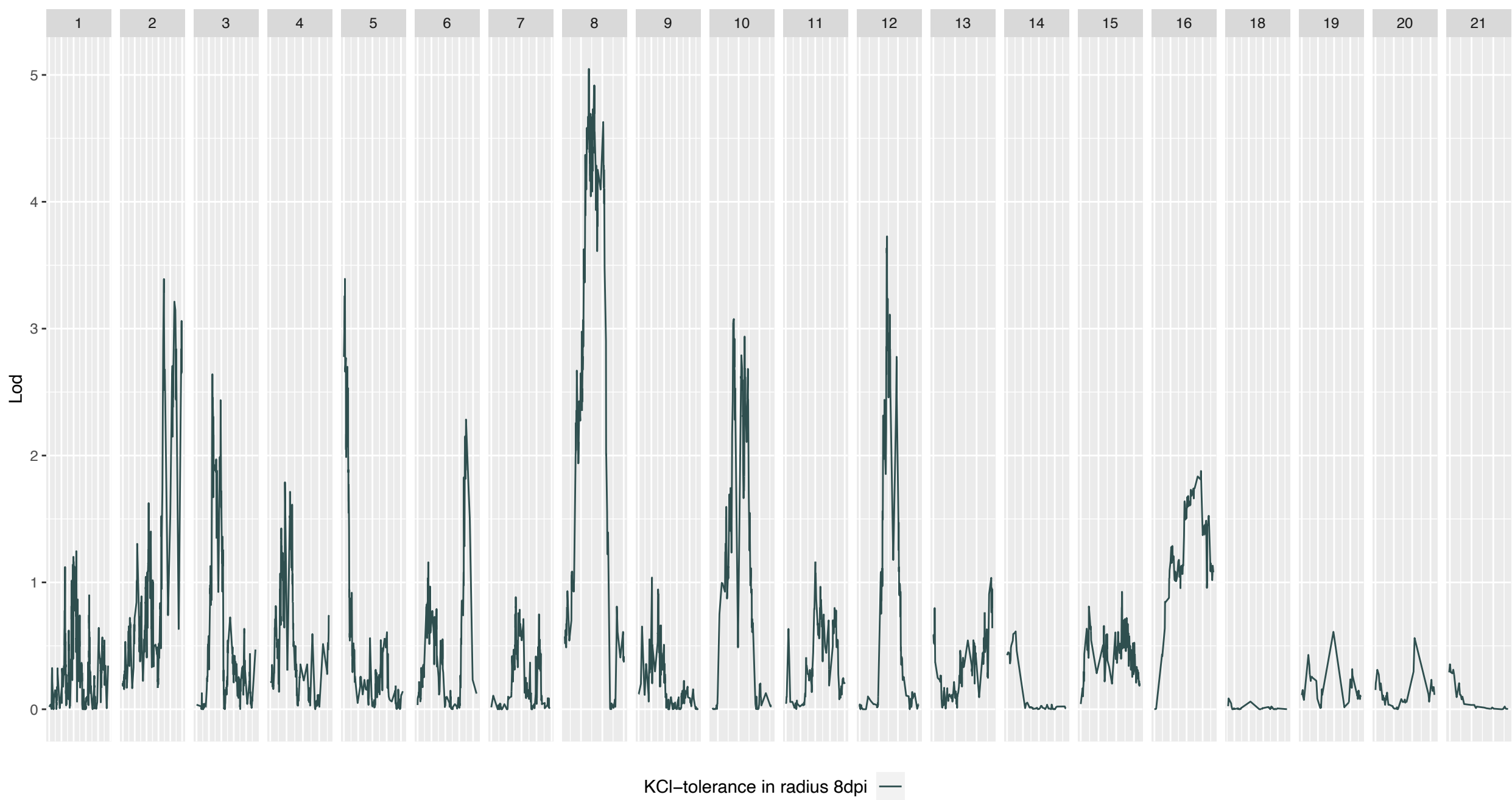

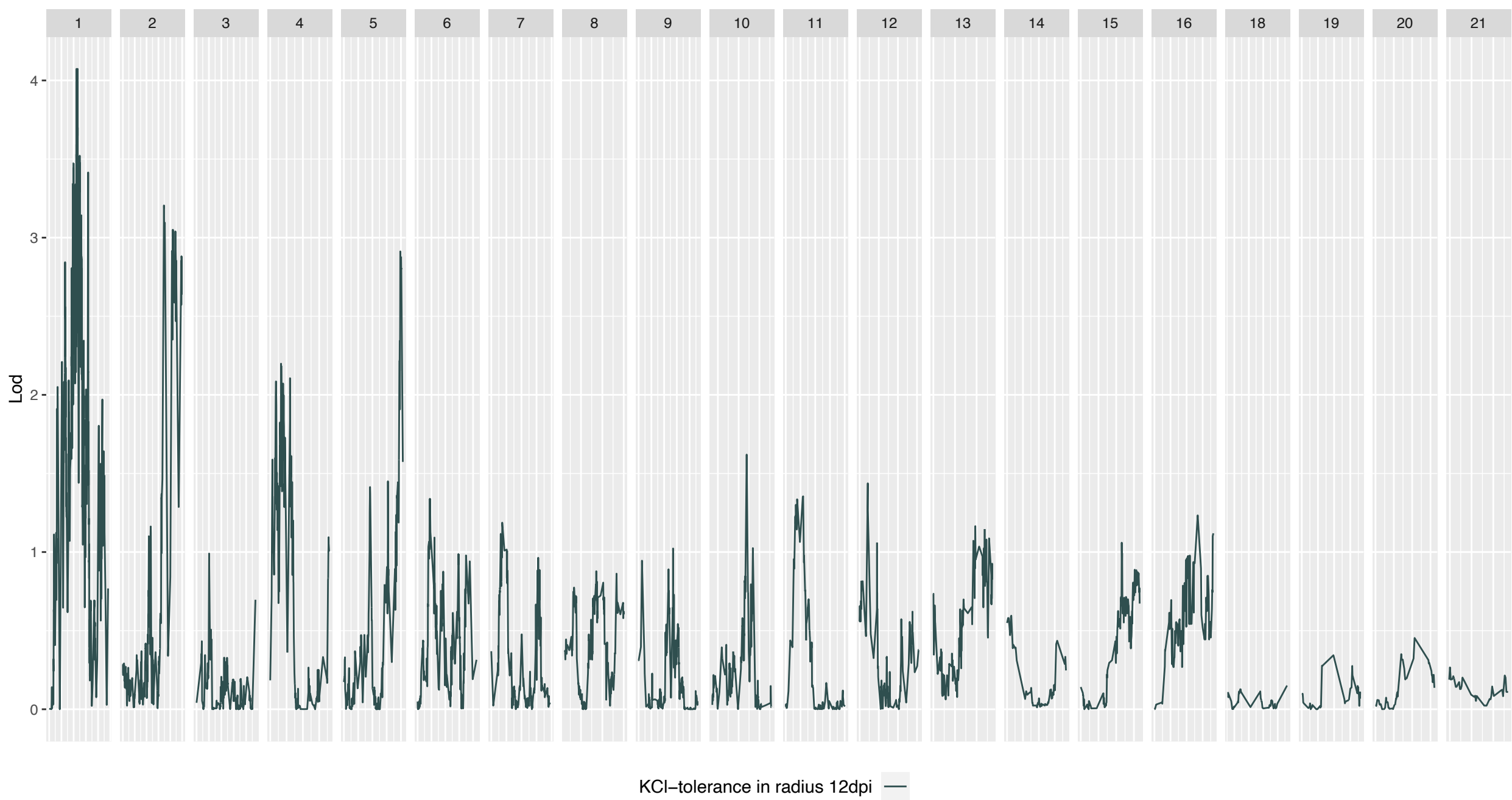

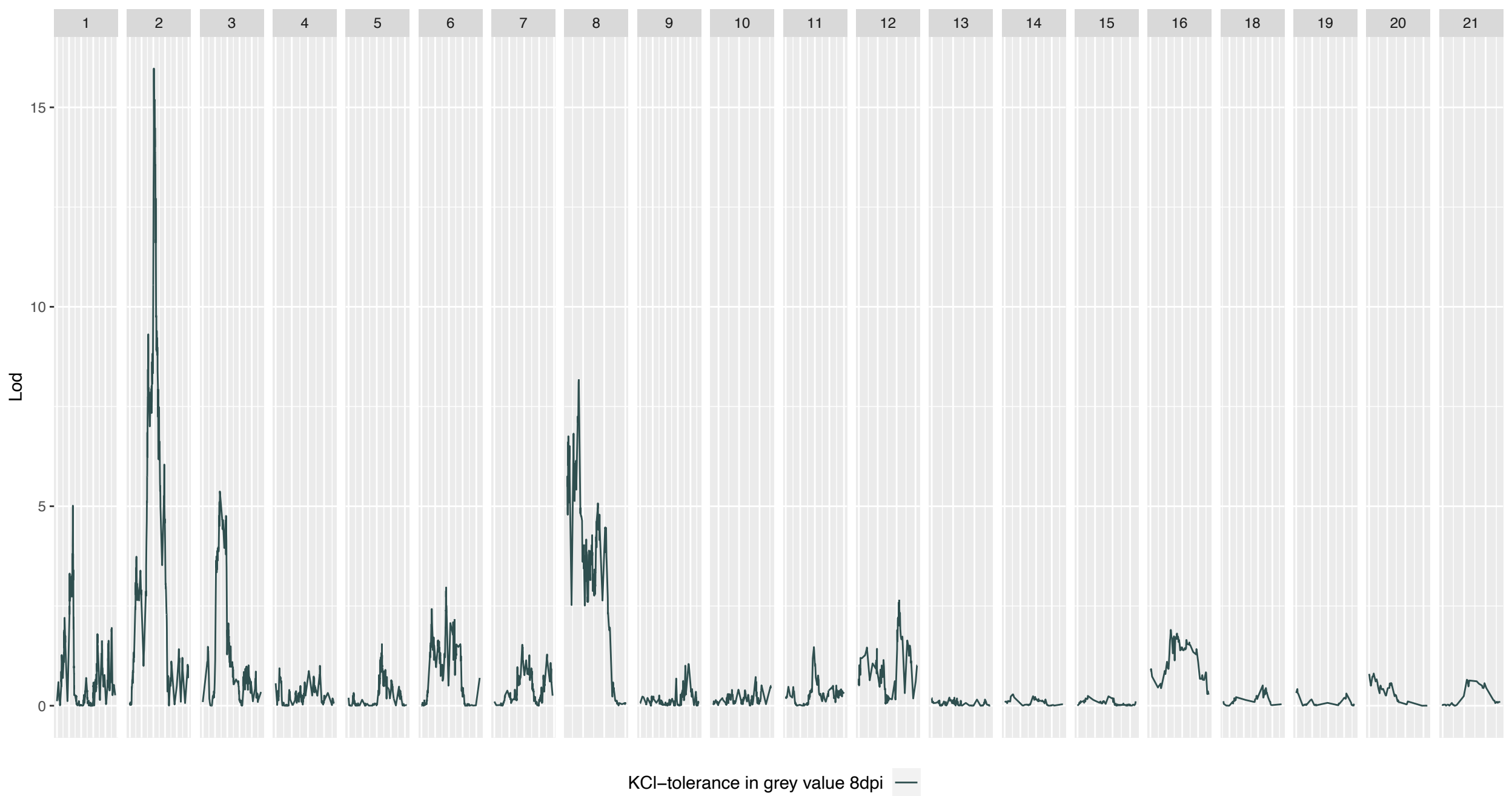

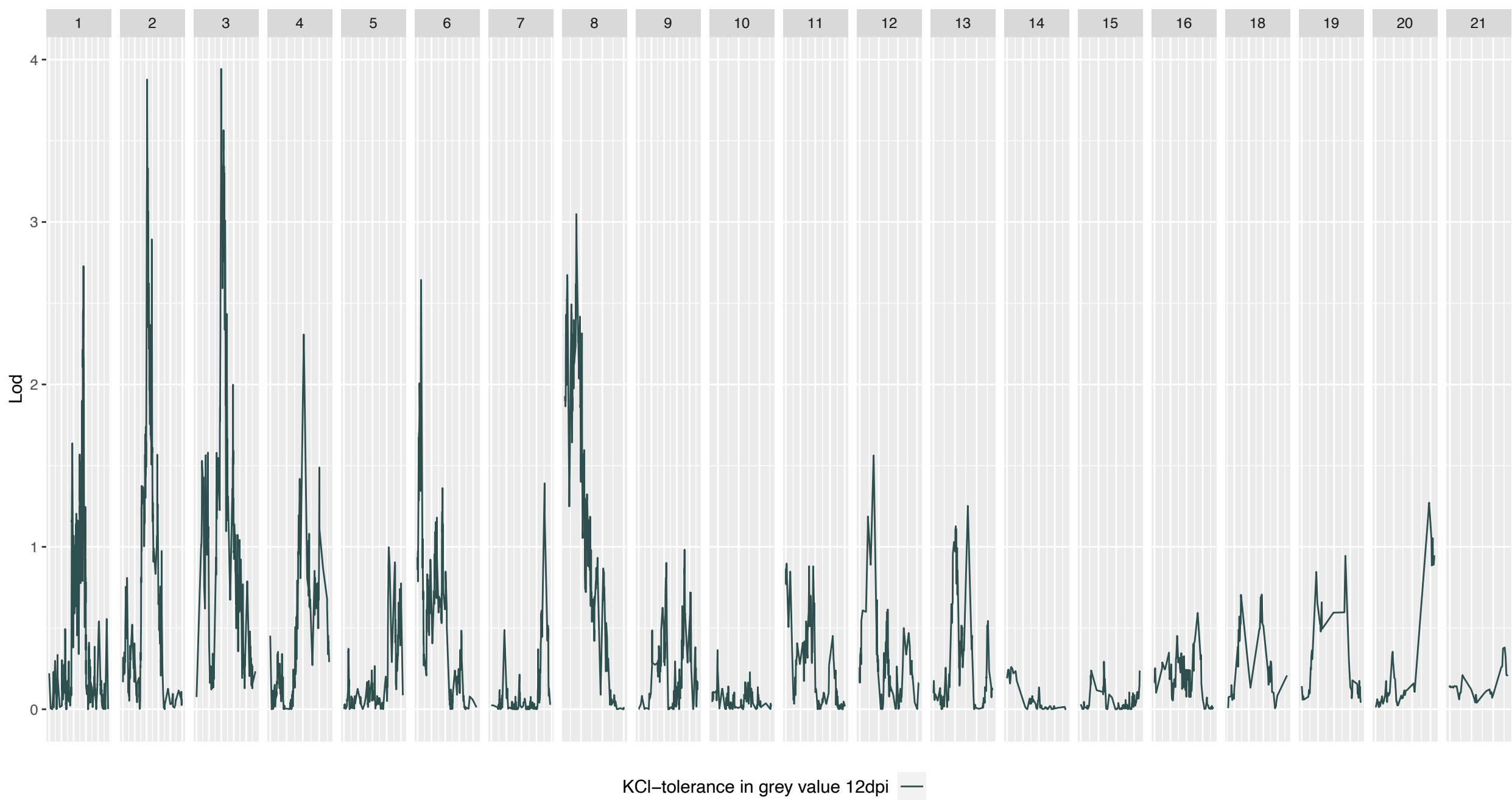

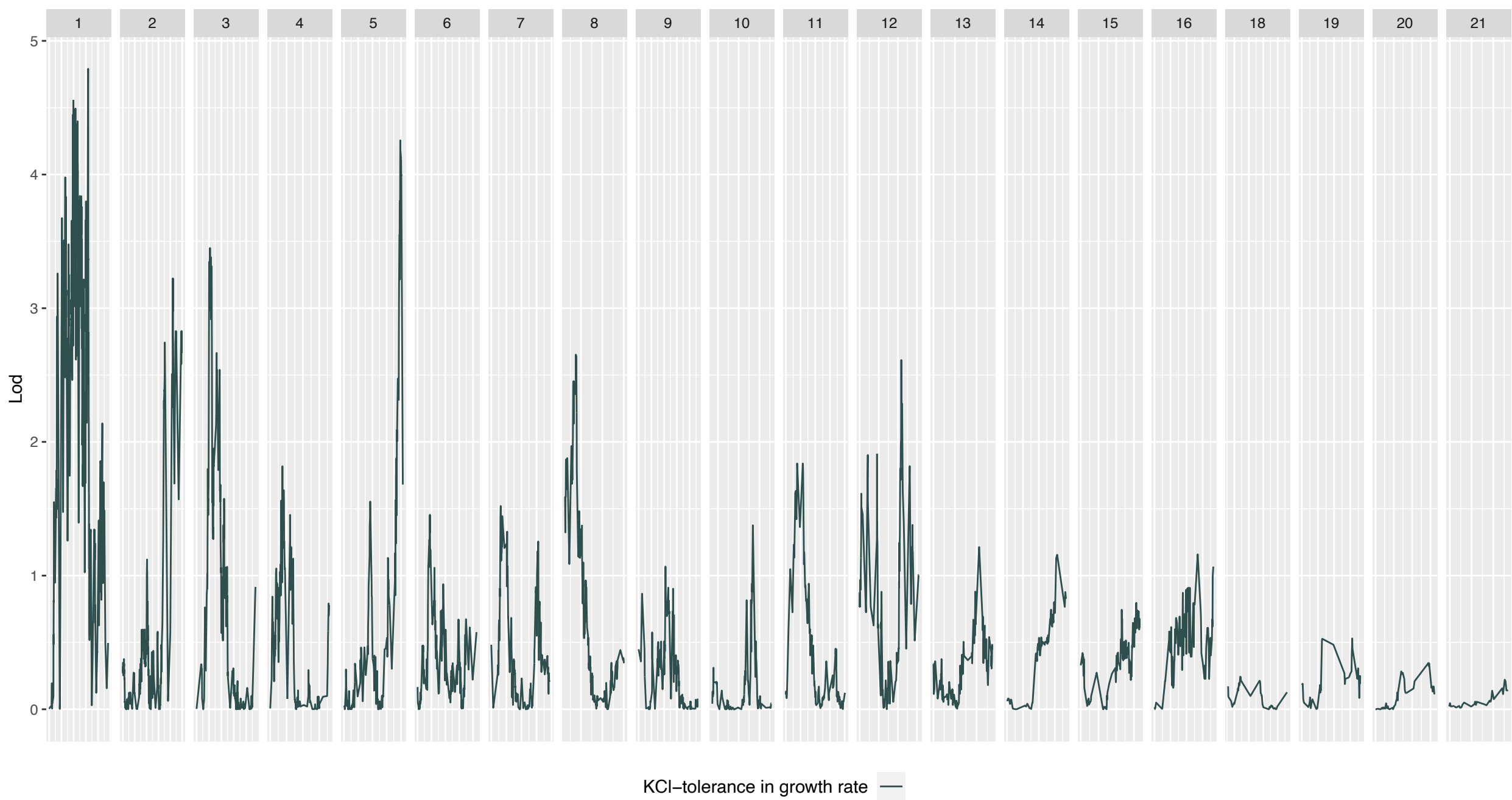

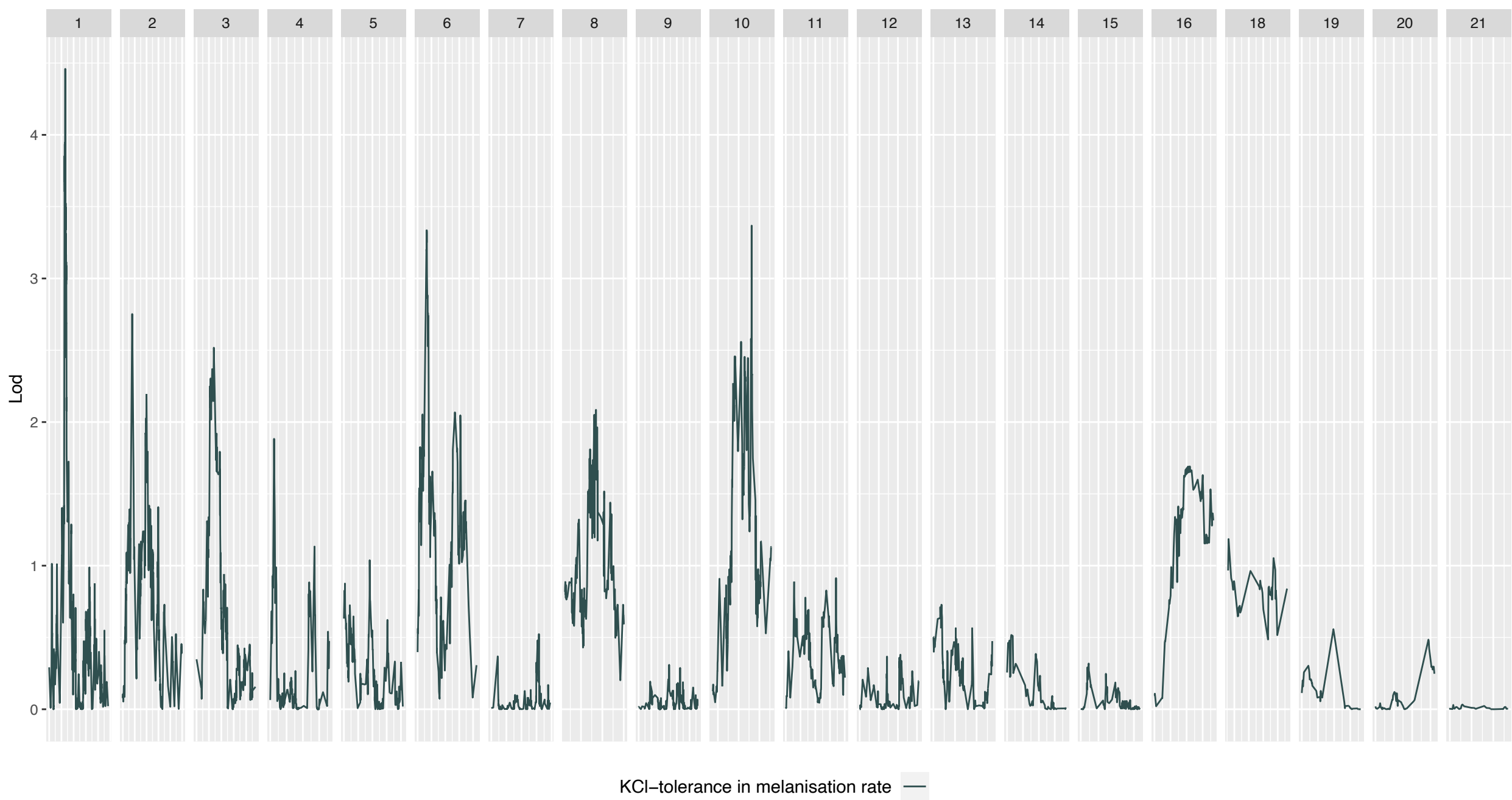

Supplement: jkad226_Supplementary_Data [file jkad226_supplementary_data.zip › Figure_S4_G3-2023-404429.pdf]
